# Exploring the Nutraceutical Potential of *Achillea millefolium* L.: Phytochemical Composition, Biological Activities, and Industrial Applications

**DOI:** 10.1002/fsn3.72002

**Published:** 2026-06-09

**Authors:** Tooba Majeed, Muhammad Tauseef Sultan, Ahmad Mujtaba Noman, Hassan Raza, Hagar M. Mohamed, Nimra Anees, Muhammad Imran, Muzzamal Hussain, Gamal A. Mohamed, Sabrin R. M. Ibrahim, Entessar Al Jbawi, Ehab M. Mostafa, Samy Selim, Ayman Salama, Mohamed A. Abdelgawad

**Affiliations:** ^1^ Department of Human Nutrition, Faculty of Food Science and Nutrition Bahauddin Zakariya University Multan Pakistan; ^2^ Department of Food Science and Technology, Faculty of Food Science and Nutrition Bahauddin Zakariya University Multan Pakistan; ^3^ Department of Medical Laboratory Analysis, College of Medical & Health Sciences Liwa University Abu Dhabi UAE; ^4^ Department of Applied Medical Chemistry, Medical Research Institute Alexandria University Alexandria Egypt; ^5^ Department of Food Science and Technology University of Narowal Narowal Pakistan; ^6^ Department of Food Sciences Government College University Faisalabad Faisalabad Pakistan; ^7^ Department of Natural Products and Alternative Medicine, Faculty of Pharmacy King Abdulaziz University Jeddah Saudi Arabia; ^8^ Department of Chemistry, Preparatory Year Program Batterjee Medical College Jeddah Saudi Arabia; ^9^ Department of Pharmacognosy, Faculty of Pharmacy Assiut University Assiut Egypt; ^10^ Sugar Beet Research Department Crop Research Administration, General Commission for Scientific Agricultural Research (GCSAR) Damascus Syria; ^11^ Department of Pharmacognosy, College of Pharmacy Jouf University Sakaka Saudi Arabia; ^12^ Pharmacognosy and Medicinal Plants Department, Faculty of Pharmacy (Boys) Al‐Azhr University Cairo Egypt; ^13^ Department of Clinical Laboratory Sciences, College of Applied Medical Sciences Jouf University Sakaka Saudi Arabia; ^14^ Department of Pharmaceutics, Faculty of Pharmacy University of Tabuk Tabuk Saudi Arabia; ^15^ Department of Pharmaceutical Chemistry, College of Pharmacy Jouf University Sakaka Saudi Arabia

**Keywords:** *Achillea millefolium* L., anticancer, antidiabetic, antioxidant potential, commercial application, pharmacological aspects

## Abstract

Medicinal plants, including *Achillea millefolium* Linn., have been used for centuries because of their health benefits. Various parts, such as flowers, leaves, and stems, are used for different purposes due to the presence of different bioactive compounds that have the potential for disease prevention and management. The present review article provides an insight into *A. millefolium* L., including its morphological description, phytochemistry, pharmacological properties, synergistic interactions with other therapeutics, nanoparticles formulation, and commercial and industrial uses. *A. millefolium* L. possesses a wide range of therapeutic applications because it can decrease oxidative stress and has strong antimicrobial activity. It is also involved in the treatment of metabolic diseases, such as diabetes, inflammation, and cancer, by modulating signaling pathways (NF‐κB, PI3K/AKT/mTOR, RAS/RAF) and inflammatory markers (IL‐6, IL‐1β, TNF‐α). The synergistic effects of *A. millefolium* L. with other medicinal plants, drugs, and nano‐formulations are important factors for its clinical applications. In addition, it has been used in commercial cosmetics, the food sector, and other products because it is considered safe and has wound‐healing properties. Finally, the wide range of phytochemicals and the essential oil composition of *A. millefolium* L. indicate broad therapeutic potential and warrant further studies into its additional benefits. Sophisticated technologies and approaches are required to design and test drug formulations and delivery systems.

## Graphical abstract

*Achillea millefolium* Linn., commonly known as yarrow, is an extraordinary medicinal plant. *A. millefolium* has broad therapeutic potential due to its phytochemistry and essential oil composition. It possesses pharmacological properties, including antidiabetic, antioxidant, anti‐inflammatory, liver‐protective, antimicrobial, and gastroprotective effects, as well as wound‐healing properties.

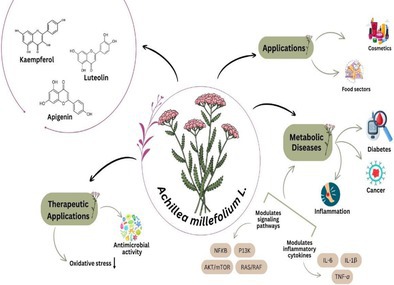

**Abbreviations**
11βHSD1: 11β‐hydroxysteroid dehydrogenase type 1
1,3‐diCQA: 1,3‐dicaffeoylquinic acid
1‐FQA: 1‐feruloylquinic acid
3,4,5‐triCQA: 3,4,5‐tricaffeoylquinic acid
3,5‐diCQA: 3,5‐dicaffeoylquinic acid
3‐CQA: 3‐caffeoylquinic acid
4,5‐diCQA: 4,5‐dicaffeoylquinic acid
4‐CQA: 4‐caffeoylquinic acid
4‐FQA: 4‐feruloylquinic acid
5‐LOX: anti‐5‐lipoxygenase activity
ABTS: 2,2'‐azino‐bis (3‐ethylbenzothiazoline‐6‐sulfonic acid
AGE: advanced glycation end products
Ag NPs: silver nanoparticles
AGS: human gastric adenocarcinoma cell line
AKT: protein kinase B
AMCSNPs: chitosan *A. millefolium* nanoparticles
AMEO: *A. millefolium* essential oil
AP‐1: activator protein 1
AsA‐GSH: ascorbate‐glutathione
BAX: Bcl‐2‐associated X protein
Bcl‐2: B‐cell Lymphoma 2
BHT: butylated hydroxytoluene
BUN: blood urea nitrogen
BW: body weight
CaOx: calcium oxalate
CAT: catalasecGMP: cyclic guanosine monophosphate
COX‐2: cyclooxygenase‐2
CYP1A2: cytochrome P450 1A2
CYP2C9: cytochrome P450 2C9
CYP3A4: cytochrome P450 3A4
CYP450: cytochrome P450
DNA: deoxyribonucleic acid
DPPH: 2,2‐diphenyl‐1‐picrylhydrazyl
DSP: daily sperm production
Dw: dry weight
EGFR: epidermal growth factor receptor
ESR: epididymal sperm reserves
FBS: fasting blood sugar
FIC: fractional inhibitory concentration
FRAP: ferric reducing antioxidant power assay
G6PD: glucose‐6‐phosphate dehydrogenase
GAK: cyclin G‐associated kinase
GAPDH: glyceraldehyde‐3‐phosphate dehydrogenase
GC/MS: gas chromatography/mass spectrophotometry
GC–FID: gas chromatography–flame ionization detector
GPx: glutathione peroxidase
GR: glutathione reductase
GSH: glutathione/reduced glutathione
HbA1C: glycated hemoglobin
HPLC: high‐performance liquid chromatography
IC50: half maximal inhibitory concentration
IL‐10: interleukin‐10
IL‐1β: interleukin‐1 Beta
IL‐6: interleukin‐6
iNOS: inducible nitric oxide synthase
KCl: potassium chloride
LC50: lethal concentration 50%
LC–MS: liquid chromatography–mass spectrometry
MAPK: mitogen‐activated protein kinase
MBC: minimum bactericidal concentration
MDA: malondialdehyde
MIC: minimum inhibitory concentration
MPO: myeloperoxidase
mRNA: messenger ribonucleic acid
MSI: mitotic segregation index
MTT assay: 3‐ (4,5‐dimethylthiazol‐2‐yl)‐2,5‐diphenyltetrazolium bromide assay
NADPH: nicotinamide adenine dinucleotide phosphate (reduced form)
NDDS: novel drug delivery system
NDV: Newcastle disease virus
NF‐κB: nuclear factor kappa B
NO: nitric oxide
OM: oral mucositis
PCOS: polycystic ovary syndrome
P‐gp: P‐glycoprotein
PI3K: phosphatidylinositol 3‐kinase
PPAR‐γ: peroxisome proliferator‐activated receptor gamma
PRF: phenolic‐rich fraction
PRF‐NLs: Phenolic‐Rich Fraction Nanoliposomes
PTLC: preparative thin layer chromatography
PTP1B: protein tyrosine phosphatase 1B
QOL: quality of life
RBC: red blood cells
SCORAD: SCORing atopic dermatitis
SGOT: serum glutamic‐oxaloacetic transaminase
SGPT: serum glutamic pyruvic transaminase
SOD: superoxide dismutase
SPF: sun protection factor
STAT3: signal transducer and activator of transcription 3
STZ: streptozotocin
TAC: total antioxidant capacity
TFC: total flavonoid content
TGL: triglycerides
TLC‐bioautography: thin layer chromatography‐bioautography
TNF‐α: tumor necrosis factor alpha
TPC: total phenolic content
UVA: ultraviolet A
UVB: ultraviolet B
VLDL: very low‐density lipoprotein
WBC: white blood cells
Zn NPs: zinc nanoparticles
ZnO: zinc oxide

## 1 Introduction

Medicinal plant‐based remedies and formulations are a key source for promoting health, preventing disease, and enhancing overall well‐being. From the dawn of humanity, people have relied on herbal remedies due to their safety, cost‐effectiveness, and availability. However, the pharmaceutical landscape of the 21st century has been revolutionized, as the adverse outcomes of these modern drugs are alarming, demanding safe approaches to manage the prevailing health problems. Thus, plants contribute significantly to minimizing the disease burden, and in recent decades, their demand has increased substantially (Khalid et al. 2025). Worldwide use of herbal medicines is growing in both developed and developing countries because they are natural alternatives and generally associated with fewer adverse effects (Raza et al. 2026). In 2017, the global herbal market was valued at USD 59.45 billion, and it is projected to reach USD 104.78 billion by 2026, with a Compound Annual Growth Rate (CAGR) of 6.5% (Bareetseng 2022). Different parts like roots, leaves, stems, and flowers of these herbs with diverse bioactive compounds are used to treat common issues (fever, cough, constipation, diarrhea) and chronic metabolic anomalies such as hypertension (HTN), diabetes mellitus (DM), cancer, cardiovascular disorders (CVDs), gastro‐pulmonary issues, hepato‐renal syndrome (HRS), and neurodegenerative diseases (Dalili et al. 2022).

The name of the genus “*Achillea*” originates from the Trojan hero Achilles, a powerful warrior in Greek mythology, and belongs to the Asteraceae family, comprising over 130 species (Ali et al. 2017). It is native to the Northern Hemisphere, from Europe to Asia, and thrives in temperate, dry climates. Among these species, *Achillea millefolium* L. is widely recognized and best known for its use in traditional medicine for over 3000 years (Jangjoo et al. 2023). *A. millefolium* L. is commonly called yarrow or milfoil in English, while in India, it has several regional names like gandana and puthkanda (Hindi), rojmaari (Marathi), achchilliya (Tamil), tukhm gandana, buiranjasif, and brinjasuf (Urdu) (Ali et al. 2017). Its wild and commercial forms are used for their nutritional and medicinal benefits. Although studies have revealed a similar chemical profile for both, there are differences between the two at the compound level. Wild samples of *A. millefolium* L. tend to contain higher levels of carbohydrates, organic acids, unsaturated fatty acids, and tocopherols. In contrast, commercial samples, purchased from a local company producing Mediterranean herbs, have higher concentrations of fats, protein, sugar, and flavonoids, which can influence their bioactivity (Dias et al. 2013). Studies have reported that. *A. millefolium* L. contains various phytochemicals including terpenoids such as camphor, monoterpenes, and sesquiterpenes. Compounds like thymol and carvacrol are present in essential oil, while flavonoids including apigenin, rutin, and lutein are found in polar extracts. Moreover, other compounds like morin, myricetin, naringin, and naringenin are also present in *A. millefolium* L. (Farasati Far et al. 2023). Sesquiterpenes and phenolic compounds contribute to the anti‐tumoral (Pereira et al. 2018), antidiabetic (Chávez‐Silva et al. 2018), antifungal (Al‐Rejaboo et al. 2024), antioxidant (Baran et al. 2025), and antimicrobial (Yeltay et al. 2025) properties of this plant.

This review provides insights into the nutritional composition, phytochemistry, and essential oil composition of *A. millefolium* L. Beyond its use in folklore, *A. millefolium* exhibits diverse pharmacological properties, including the modulation of oxidative stress (Shaiea et al. 2024) and metabolic regulation (Rezaei et al. 2020), among others. In addition to its health‐promoting properties, the review also discusses the plant's potential applications in the food and cosmetics industries. Furthermore, it highlights the synergistic and comparative analyses of *A. millefolium* L. with other medicinal plants, its interaction with drugs, and its role in nanoparticle‐based formulation. Overall, it offers a comprehensive perspective on *A. millefolium* across disciplines, making this review a distinctive and valuable resource.

## 2 Research Methodology

The data for this review were collected from various databases, including Google Scholar, PubMed, and ScienceDirect. Several keywords, such as *A. millefolium* L., yarrow, chemical composition, therapeutic effects, antidiabetic effects, health benefits, medicinal properties, antioxidant effects, antimicrobial effects, pharmacological aspects, gastroprotective effects, synergistic effects, drug interactions, commercial use, and industrial use, were used to search the data. This review examines the therapeutic properties of *A. millefolium* L. by compiling relevant data from the past 25 years (2000–2025). While the majority of studies in this review are recent, some earlier publications were also considered. Inclusion criteria included studies on *A. millefolium* L. chemical composition, health benefits, nanoparticles development, in vivo and in vitro trials, and commercial and industrial applications. Studies other than *A. millefolium* L. and articles in languages other than English were excluded.

## 3 Morphological Description and Geographical Distribution

**FIGURE 1:**
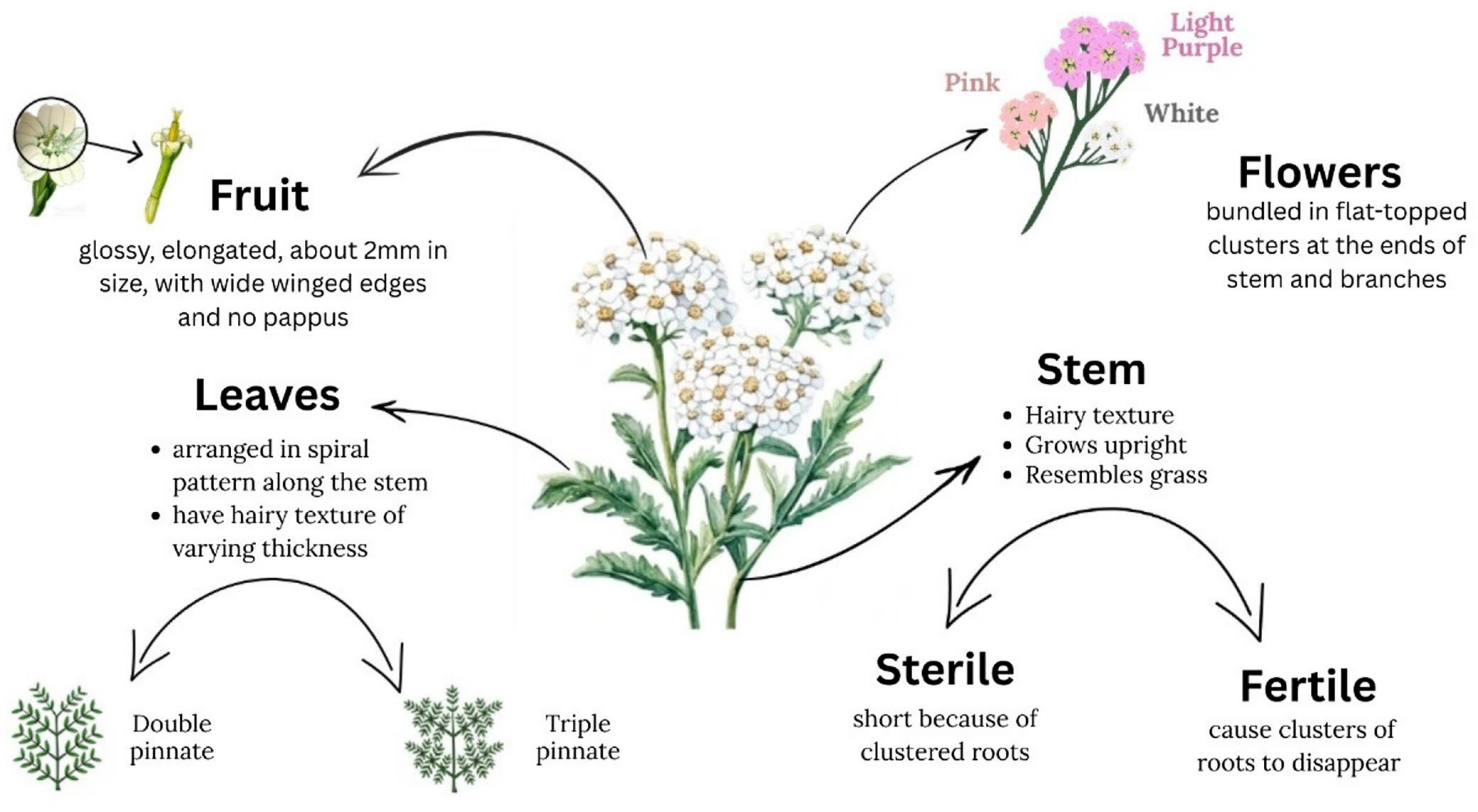
Botanical and Morphological Depiction.

**FIGURE 2:**
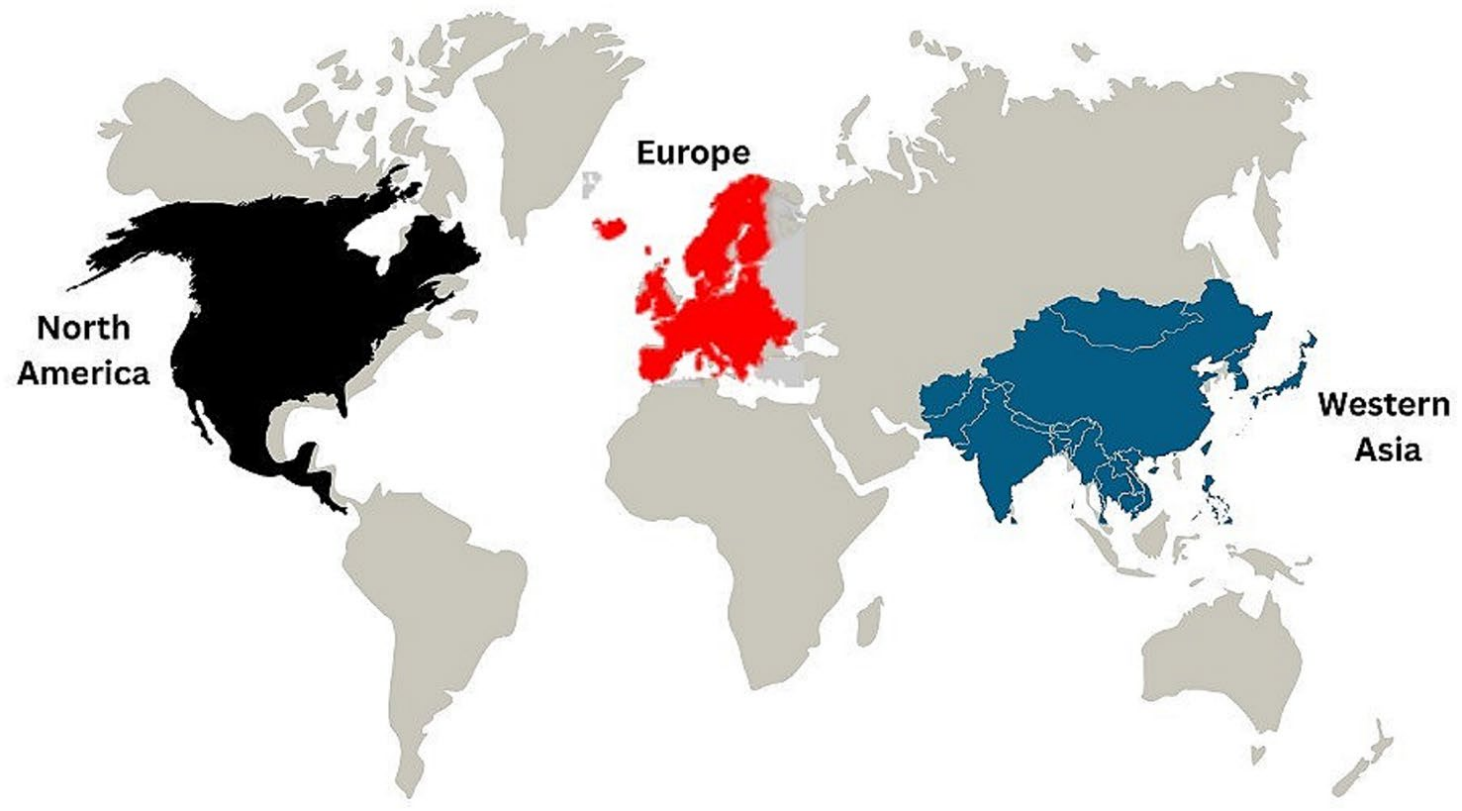
Geographical Distribution.

*Achillea millefolium* L. is a long‐lasting herbaceous plant with light brown roots. In the vegetative phase, its stem appears short due to the presence of clustered roots. Stems are of two types: one is sterile, growing horizontally under the soil to nourish the plant, and the other, called fertile, appears during the plant's reproductive cycle, causing the cluster of roots to disappear as the nodes separate. In short, the stems have a hairy texture, grow upright, and resemble grass (Ilham et al. 2022). The leaves of *A. millefolium* L. are either double or triple pinnate, arranged in a spiral pattern along the stem. Hairy textures with varying thickness cover the leaves (Ali et al. 2017). Leaves of this species resemble those of ferns, as they have a feathery texture (Ayoobi et al. 2017). Mostly, morphology studies focus on vegetative organs, as it is difficult when the plant enters its reproductive stage (Ilham et al. 2022). The flowers can be of various colors, such as white, pink, or light purple, and are bundled in flat‐topped clusters at the ends of stems and branches. The fruits are glossy, elongated achenes, approximately 2 mm in size, with wide‐winged edges and no pappus (Akram 2013). *A. millefolium* L. is distributed all over the world, but for the most part, it is found in Europe and western Asia, grown in temperate regions, including North America. It is typically found in meadows and open forests at altitudes of up to 3500 m. Most active growth of this plant occurs in the spring season and blooms from May to June (Jangjoo et al. 2023). Figure 1 illustrates the botanical and morphological depiction of *A. millefolium* L. Figure 2 shows the geographical distribution of *A. millefolium* L.

## 4 Taxonomical Classification

As per the United States Department of Agriculture's (USDA) “Plant Database,” *A. millefolium* L. belongs to the domain Eukarya, kingdom Plantae, which consists of multicellular organisms that perform photosynthesis. The phylum is Anthophyta, which includes flowering plants whose seeds are enclosed within their fruits. It is classified under the class Magnoliopsida. The order of *A. millefolium* L. is Asterales, in which the ovary is located below the flowers, and flowering heads are arranged in a spiral pattern. Within this order, *A. millefolium* L. belongs to the family Asteraceae, in which many small flowers are clustered together. It belongs to the genus *Achillea*, which is named after the Greek warrior Achilles. The species name is *millefolium* (Bashir et al. 2022).

## 5 Primary Metabolites and Mineral Nutrients in *A. millefolium* L.

A study investigated the nutritional composition of both commercial and wild samples of *A. millefolium* L. According to the results, the commercial sample contained 63.90 ± 0.86, 8.03 ± 0.00, 19.53 ± 0.05, and 8.54 ± 0.88 g/100 g dw of carbohydrates, fat, protein, and ash, respectively. In contrast, the wild sample (inflorescences and upper leaves) contained 75.84 ± 0.76, 5.20 ± 0.13, 12.53 ± 0.85, and 6.43 ± 0.11 g/100 g dw of carbohydrates, fat, protein, and ash (Dias et al. 2013). Another study reported that the dry matter content in *A. millefolium* L. was 92.11%, comprising 9.57% crude protein, 31.98% crude fiber, 3.37% ether extract (fat), and 39.14% carbohydrate content in the form of nitrogen‐free extract (Biel et al. 2023). One study also reported the physicochemical characterization of *A. millefolium* L. aerial parts and found that the moisture content was 9.50% ± 0.40%, while the total ash content was 8.03% ± 0.23%. Similarly, acid‐insoluble ash content, water extractive value, and methanol extractive value were 1.33% ± 0.25%, 18.50% ± 1.35%, and 6.47% ± 0.31%, respectively (Kumar et al. 2021). *A. millefolium* L. was also found to be rich in elements like potassium (2.43%), calcium (2.22%), nitrogen (1.01%), magnesium (0.70%), phosphorus (0.63%), while other elements such as iron (360.4 mg/kg), manganese (85.5 mg/kg), zinc (47.6 mg/kg), and copper (28.3 mg/kg) were also present in it (Saraç et al. 2021).

## 6 Secondary Metabolites in *A. millefolium* L

**FIGURE 3:**
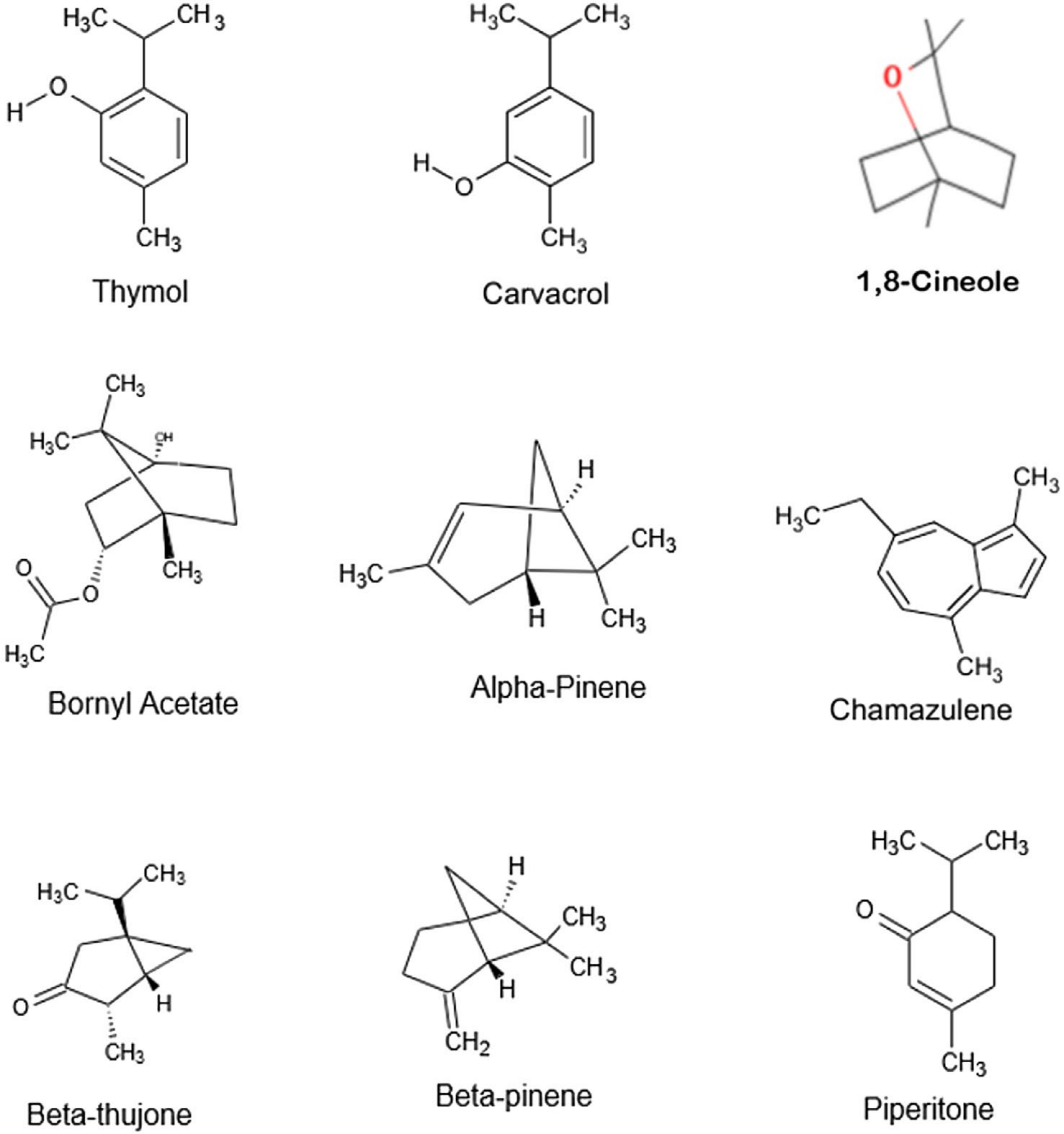
Major phytochemicals in the essential oil of *Achillea millefolium* L.

The phytochemicals present in the medicinal plants are the reason behind their therapeutic potential. These phytochemicals can be categorized according to their metabolic activity. Primary metabolites include amino acids, sugars, proteins, and chlorophyll, among others. However, alkaloids, flavonoids, saponins, phenolic compounds, and others are considered secondary metabolites (Agidew 2022). The major phytochemicals of *A. millefolium* L. are presented in Figure 3.

A study conducted in Azad Jammu and Kashmir, Pakistan, reported that the ethanolic extract of *A. millefolium* L. contained bioactive components, including flavonoids, alkaloids, phenols, saponins, carbohydrates, coumarins, and phlobatannins (Gorayah et al. 2023). Essential oils (EOs) of plants are known to contain diverse bioactive components. Essential oils are low molecular weight, volatile substances that are produced in plant parts (flowers, leaves, stems, roots, seeds, etc.). Essential oils are composed of various aromatic and bioactive compounds, such as volatile oils, flavonoids, and phenols (Bolouri et al. 2022); however, terpenes and their derivatives, such as monoterpenes and sesquiterpenes, are dominant components which constitute the major portion of essential oils (Ramsey et al. 2020). However, the composition, yield, and bioactivity of essential oils are influenced by various factors, including genetic factors, species, ecotype, chemotype, variety, and the part of the plant used. Moreover, environmental conditions, cultivation methods, harvesting, extraction techniques, storage conditions, and testing methods not only influence the oil quality but also affect the stability and activity of oils (Abdelmohsen and Elmaidomy 2025). Essential oils extraction can be done through diverse techniques, including traditional methods like steam and hydro‐distillation, to advanced approaches such as microwave‐assisted extraction and supercritical CO_2_ extraction. These methods play a crucial role in determining the oil's yield, composition, and quality, as inappropriate procedures can alter its chemical integrity (Mugao 2024).

One study assessed the variation in the composition of the essential oil of aerial parts of four populations of yarrow collected from different altitudes of Dagestan. The oil yield ranged from 0.06 to 0.016 mL/100 g, and a total of 61 compounds were identified. Major compounds were monoterpenoids such as α‐pinene, sabinene, and camphene (Radzhabov et al. 2022). Moreover, another investigation on the essential oil composition of *A. millefolium* L. identified 42 major compounds. Oxygenated monoterpenes made up 58.95% of the oil. Key compounds present in the oil were piperitone (12.79%), p‐cymene (10.60%), and bornyl acetate (8.58%) (Ebadollahi et al. 2016). In another study, it was found that mature yarrow leaves yield 0.46% essential oil. They discovered that the main components were borneol (35.9%) and camphor (18.8%) in EO (Gharibi et al. 2015). The composition of the essential oil from the flowering aerial parts of *A. millefolium* L. (East Azerbaijan, Iran) was also studied, and it was found that the oil yield was 0.35%. Major components were 1,8‐cineole (28.0%) and camphor (19.2%), among a total of 20 compounds (Dehghan and Elmi 2015). In another study, the essential oil of flowers and leaves was also investigated, and it was found that the yield of oil from flowers and leaves was 0.03% for an 80 kg plant. A total of 11 volatile compounds were identified, with β‐thujone being the dominant compound (96.2%) (Tampe et al. 2015).

According to one study conducted in the Lublin region of eastern Poland, the volatile essential oil composition of *A. millefolium* L. flowers was analyzed using SPME‐GC/MS, while GC/MS was employed to evaluate the total oil content of fresh and dried (stored) flowers. SPME‐GC/MS revealed that floral volatiles are mainly composed of monoterpenes, including α‐pinene (7.1%), 1,8‐cineole (6.6%), β‐pinene (3.7%), along with sesquiterpenes such as germacrene D (18.2%), β‐caryophyllene (11.1%), as well as trace n‐alkanes. GC/MS analysis showed variations in the EO composition of fresh and stored flowers. Fresh flowers contained relatively higher proportions of monoterpenes like α‐pinene (18.5%), sabinene (8.8%), whereas stored samples showed an increased abundance of sesquiterpenes, including β‐caryophyllene (6.3%), (E)‐nerolidol (4.1%) and β‐caryophyllene oxide (3.9%). Notably, chamazulene (12.6%) was only detected in stored flowers (Paduch et al. 2008). Orav et al. (2006) reported the essential oil composition of commercial *A. millefolium* L. plant materials from different European countries. Samples from Estonia, Greece, Germany, Lithuania, Moldavia, Latvia, and Hungary contained higher proportions of monoterpenes (12.4%–36.8%) and sesquiterpenes (31.1%–53.1%), particularly β‐pinene and (E)‐β‐caryophllene. In contrast, oils from Armenia, Spain, Russia, France, Italy, and Belgium were dominated by oxygenated monoterpenes (53.9%–76.1%) such as α‐thujone, β‐thujone, and camphor. Additionally, samples from countries like Scotland, Ukraine, and Ural showed higher levels of oxygenated sesquiterpenes (21.5%–32.3%) including α‐bisabolol, β‐bisabolol, and caryophyllene oxide. Essential oil composition of white, pink, and deep pink yarrow flowers was also investigated in Vilnius, Lithuania. As a result, inflorescence oil yield ranged from 0.8% to 1.0%, and leaf oil yield ranged between 0.2% and 0.4%. The main components in the inflorescence oil were β‐pinene (19.3% and 18.0%) in white and pink flowers, and (E)‐nerolidol (16.1%) in deep pink flowers. In leaf oil of white, pink, and deep pink flowers, β‐eudesmol (12.8%), borneol (10.7%), and piperitone (10.8%) were major components. Monoterpenes (23.8%–35.4%) and sesquiterpene hydrocarbons (8.3%–14.5%) were higher in flower oils, while oxygenated sesquiterpenes (41.0%–47.0%) were higher in leaf oils (Judzentiene and Mockute 2005). Dokhani et al. (2005) also reported that *A. millefolium* L. flowers collected from different locations in Iran had higher concentrations of volatile compounds as compared to leaves. Compounds like α‐pinene, β‐pinene, 1,8‐cineole, and sabinene were the dominant ones. Similarly, flowers also had more total phenolic and tartaric contents than leaves. Saeidnia et al. (2004) conducted a comparative study to analyze the essential oil composition of *A. millefolium* L. and *A. talagonica*. Major components in *A. millefolium* L. were α‐copaene (11.1%) and (E)‐nerolidol (8.8%), while 1,8‐cineole (9.7%) and camphor (21.9%) were the main ones in *A. talagonica*.

In addition to the phytochemical composition of *A. millefolium* L. essential oils, several studies have also investigated its other bioactive compounds. Gharibi et al. (2015) reported that the total phenolic content (TPC) and total flavonoid content (TFC) in the methanolic extract of *A. millefolium* L. leaves were 47.20 mg tannic acid/g weight and 66.37 mg quercetin/g dry weight, respectively. Similarly, a study on phenolic compounds in *A. millefolium* L. plant resulted in the presence of chlorogenic acid (458.001 μg/g), caffeic acid (41.797 μg/g), salicylic acid (39.285 μg/g) in higher amounts along with moderate amounts of trans‐ferulic acid (21.151 μg/g), trans‐cinnamic acid (20.679 μg/g), quercetin (16.736 μg/g), and naringenin (15.687 μg/g) (Baran et al. 2025). According to Topatan et al. (2024), extraction of upper leaves and inflorescences of *A. millefolium* L. with methanol/dichloromethane revealed major phenolic compounds including rutin (112.355 μg/g), caffeic acid (76.232 μg/g), and vanillin (27.063 μg/g), while quercetin (11.856 μg/g), 4‐OH benzoic acid (9.448 μg/g), salicylic acid (9.433 μg/g), p‐coumaric acid (4.177 μg/g), sesamol (4.083 μg/g), syringic acid (2.805 μg/g), and rosmarinic acid (2.410 μg/g) were present in moderate to low amounts. A comparative study of aerial parts (inflorescences, leaves, and stems) from Turkey (Gaziantep, southeastern Anatolia; Nevşehir, Central Anatolia) and Lithuania (wild population) identified 20 compounds, including 11 flavonoids (quercetin, rutin, quercitrin, isoquercitrin, santin, luteolin, apigenin, and their glycosides) and 9 phenolic acids (chlorogenic acid, neochlorogenic acid, caffeic acid, and caffeoylquinic acid derivatives). Chlorogenic acid was the most abundant compound, present in higher levels in leaves (19,483 vs. 4140.3 μg/g), inflorescences (7735 vs. 1443.8 μg/g), and stems (4069.3 vs. 1068.6 μg/g) of Lithuanian samples as compared to Turkish samples (Radušienė et al. 2023). Furthermore, a study also reported the presence of protocatechuic acid, caffeic acid, 1,3‐diCQA, 1‐FQA, 4‐CQA, 3‐CQA, 3,5‐diCQA, 4,5‐diCQA, and 3,4,5‐triCQA in the methanol, ethyl acetate, and water extracts of *A. millefolium* L. aerial parts collected at the flowering stage. However, 4‐FQA was detected only in the methanolic extract (Zengin et al. 2017). Qureshi et al. (2016) also quantified flavonoids in hydrolyzed *A. millefolium* L. plant extract and reported the presence of total polyphenolic compounds (68.84 mg/g), apigenin (0.04833 mg/mL), luteolin (0.03831 mg/mL), quercetin (0.01202 mg/mL), isorhamnetin (0.00117 mg/mL), and kaempferol (0.00046 mg/mL). Beyond these compounds, *A. millefolium* L. also contains sesquiterpene lactones. A study in China isolated eight germacrane‐type sesquiterpene lactones in the ethyl acetate fraction of *A. millefolium* L. whole plant. These included six new compounds (millefoliumons A–F) along with two known compounds such as tans, trans‐germacra‐1 (10),4‐dien‐6α,12‐olide and 3β‐hydroxy‐11αH‐germa‐1 (10),4‐dien‐12,6α‐olide (Li et al. 2023). In another study in China, a total of 12 compounds were isolated from the whole plant of *A. millefolium* L. extract (95% EtOH and 50% EtOH) and its fractions. Among them, seven were newly identified guaianolide sesquiterpene lactones, namely Millefoliumins (A–G) while the remaining five known compounds included 8α‐angeloxy‐1β,2β,4β,5β‐diepoxy‐10β‐hydroxy‐6Βh,7αH,11βH‐12,6α‐guaianolide, 8α‐angeloxy‐4α,10β‐dihydroxy‐2‐oxo‐6βH,7αH,11βH‐1 (5)‐guaien‐12,6α‐olide, austricin, matricarin, and malaphyllidin (Li et al. 2021). Moreover, the ethyl acetate fraction of the whole plant was found to contain three new sesquiterpenes, namely sesquiterpene lactone‐esters (A and B) and a lactone‐diol (Farooq et al. 2012). Collectively, the phytochemical profile of *A. millefolium* L. shows high chemical diversity across essential oils, phenolics, and sesquiterpene lactones. Essential oil studies reveal considerable variability in yield and composition, mainly consisting of monoterpenes and sesquiterpenes. In contrast, phenolic and flavonoid analyses indicate the presence of key compounds such as chlorogenic acid, quercetin, caffeic acid, luteolin, apigenin, and others across different extracts. Table 1 summarizes the chemical composition of *A. millefolium* L. essential oil extracted from aerial parts at different growth stages and locations.

**TABLE 1:** Volatile and non‐volatile chemical profile of *A. millefolium* L.

Location	Parts and forms	Techniques	Phytochemicals	References
Croatia	Aqueous herbal infusion (whole plant)	HPLC–PDA	Total phenolic acids (648.13 ± 5.47 mg/L) Chlorogenic acid and its derivatives (337.38 ± 4.72 mg/L) Ferulic acid and its derivatives (189.66 ± 4.23 mg/L) Total flavonoids (174.74 ± 1.97 mg/L) Apigenin and its derivatives (72.84 ± 0.95 mg/L) Luteolin and its derivatives (56.02 ± 0.48 mg/L)	Bilušić et al. (2025)
Iran	Flowering branches (aqueous extract)	HPLC	Apigenin (1.6 ± 0.12 mg/g) Luteolin (0.3 ± 0.01 mg/g)	(Moradi et al. 2024)
Pakistan	Fresh parts (roots, stem, leaves) Methanol and chloroform extract	Gravimetric method, Folin–Ciocalteu colorimetric method, spectrophotometric method	Methanol extract: high amount of phenols (34.5%), saponins (31.6%), alkaloids (27.6%), tannins (26.5%) Chloroform extract: high amount of terpenoids (35.5%) and flavonoids (32.6%)	(Adil et al. 2024)
Pakistan	Fresh parts (roots, stem, leaves) Methanol and chloroform extract	GC–MS	Methanol extract: octadecanal (6.21%), ethanone, 1‐(4‐hydroxy‐3, 5‐dimethoxyphenyl) (5.35%), ergost‐5‐en‐3‐ol, (3.beta.) (3.85%) Chloroform extract: n‐hexadecanoic acid (10.6%), squalene (5.43%), 9,12,15‐octadecatrienoic acid (3.67%)	(Adil et al. 2024)
Uzbekistan	Aerial parts EO	GC–MS	2‐karen (20.60%), ascaridol (10.06%) and benzene, 1‐methyl‐3‐(1‐methylethyl)–(8.78%), monoterpenes and sesquiterpenes	(Istamkulova et al. 2023)
Turkey	Aerial Parts EO	GC–FID and GC–MS	Oxygenated sesquiterpenes (29.6%), oxygenated monoterpenes (25.17%), 1,8‐cineole (12.3%), β‐eudesmol (8.9%), α‐pinene (4.3%), caryophyllene oxide (4.2%), β‐pinene (2.9%)	(Toplan et al. 2022)
Turkey	Aerial parts methanolic extract	LC–MS/MS	Quinic acid, malic acid, chlorogenic acid derivatives, protocatechic acid hexoside, quercetin glycoside, quercetin rutinoside, luteolin glycoside, apigenin glucoside, apigenin, luteolin	(Toplan et al. 2022)
Romania	Flowers Hydro‐alcoholic extract	Colorimetric and spectrophotom‐etric method	Flavonoids (rutin) 0.24% g/g, polyphenolic carboxylic acid (caffeic acid) 0.0625% g/g, triterpenic saponins 0.5%	(Grigore et al. 2020)
Iran	Aerial parts EO (flowering, vegetative and fruit stage)	GC–MS, GC–FID	1,8‐cineole (21.28%–34.51%), camphor (7.27%–14.07%), chamazulene (4.18%–11.34%), α‐eudesmol, α‐cadinol, borneol	(Farhadi et al. 2020)
Iran	Aerial parts EO (various accessions)	GC–MS	b‐thujone (0.4%–55.3%), transnerolidol (0.4%–48.1%), camphor (0.6%–25.5%), 1, 8‐cineole (1.2%–19.8%), isospathulenol, and cubenol	(Farajpour et al. 2017)
France	Aerial parts EO	GC–MS	Oxygenated monoteprenes (40.7%) Camphor (12.8%), trans‐chrysantenyl acetate (6.6%), terpinen‐4‐ol (4.70%), (E)‐p‐mentha‐2,8‐dien‐1‐ol (4.5%), 1,8‐cineole (4.0%) Oxygenated sesquiterpenes (19.5%) (E)‐nerolidol (7.3%), Hydrocarbon sesquiterpenes (17%) germacrene‐D (12.0%) Monoterpenes (15%) sabinene (6.7%), β‐pinene (3.4%)	(El‐Kalamouni et al. 2017)
Italy	Aerial parts methanolic extract	TLC, RP‐HPLC, HPLC–DAD, HPLC–ESI‐MS, ^1^H‐NMR	Chlorogenic acid, rutin, luteolin 7‐O‐glucoside, 1,3‐dicaffeoylquinic acid, 1,4‐dicaffeoylquinic acid, 3,4‐dicaffeoylquinic acid, apigenin 4′‐O‐glucoside, apigenin 7‐O‐glucoside, luteolin 4′‐O‐glucoside, 3,5‐dicaffeoylquinic acid	(Vitalini et al. 2011)
India	Aerial parts EO	GC–MS	Sabinene (17.58%), 1,8‐cineole (13.04%), borneol (12.41%), bornyl acetate, alpha‐pinene, beta‐pinene	(Nadim et al. 2011)
Southern Siberia	Aerial parts EO	LC–MS	Oxygenated compounds (50.9%), sesquiterpenes (11.1%), 1, 8‐cineole (28.8%), and others.	(Smelcerovic et al. 2010)
Lithuania	Flowers (hydro‐ethanolic extract)	HPLC	Chlorogenic acid (2.83–12.67 mg/g), luteolin‐7‐O‐glucoside (2.10–5.88 mg/g), apigenin7‐O‐glucoside (3.334–7.546 mg/g), luteolin, and apigenin	(Benetis et al. 2008)
Turkey	Aerial parts EO	GC–MS	Eucalyptol (24.6%), camphor (16.7%), α‐terpineol (10.2%), β‐pinene (4.2%), borneol (4.0%)	(Candan et al. 2003)
India	Aerial parts EO (flowering stage)	GC, GC–MS	Camphor (28%), 1, 8‐cineole (12%), germacrene‐D (12%), cis‐chrysanthenyl acetate (8%)	(Shawl et al. 2002)

Abbreviations: ^1^H NMR, proton nuclear magnetic resonance; EO, essential oil; GC, gas chromatography; GC–FID, gas chromatography–flame ionization detector; GC–MS, gas chromatography–mass spectrometry; HPLC, high‐performance liquid chromatography; HPLC–DAD, high‐performance liquid chromatography with diode array detector; HPLC–ESI‐MS, high‐performance liquid chromatography–electrospray ionization‐mass spectrometry; HPLC–PDA, high‐performance liquid chromatography with photo diode array detector; LC–MS, liquid chromatography–mass spectrometry; TLC, thin layer chromatography.

## 7 Pharmacological Properties

Various medicinal plants worldwide fall under the genus *Achillea*. Their traditional uses can vary from culture to culture and from one region to another. However, in general, these species are used to treat different diseases and complications in the form of infusions or decoctions made from herbs, flowers, and leaves. These remedies aid in the treatment of hemorrhages, liver disorders, gastrointestinal complications, and inflammatory conditions and other ailments (Barda et al. 2021).

Pharmacological research conducted worldwide has demonstrated that *A. millefolium* L. exhibits therapeutic effects in treating various inflammatory and infectious conditions. Moreover, these studies have also confirmed that the plant has antimicrobial, antiviral, and antitumor activities (Tripon et al. 2024). Several chemical constituents, including components of essential oils such as sesquiterpenes and phenolic compounds, present in the plant, are responsible for its pharmacological properties. Moreover, phenols and flavonoids are responsible for yarrow's antibacterial activity. Hexane, methanol, and petroleum ether extracts from the aerial parts were found to be active against *Staphylococcus aureus* , *Escherichia coli* , *K. pneumoniae* , *A. niger* , *C. albicans* , and other microorganisms (Grigore et al. 2020). *A. millefolium* L. has also demonstrated a wide range of pharmacological activities, including hepatoprotective activity (Daneshvar‐Ghahfarokhi et al. 2025). Additionally, it has also been used in the treatment of hemorrhoids (Mahmoudi et al. 2023) and menstrual disorders (Radfar et al. 2019). Table 2 showed the pharmacological properties of *A. millefolium* L.

**TABLE 2:** Pharmacological Properties of *A. millefolium* L.

Pharmacological properties	Part/Form used	Assay/Model	Dose (s)	Effective Dose/Compounds/key findings	References
Cytotoxic	Flowering tops (herbs) (methanol and dichloromethane extract)	In vitro (on normal fetal lung fibroblasts MRC‐5, non‐small cells lung adenocarcinoma A549, colorectal carcinoma epithelia cells HCT 116)	0.25, 0.5, 1, 2, and 4 mg/mL	4 mg/mL Dichloromethane extract: ↓ MRC‐5 survival (92%), ↓ A549 survival (94%), ↓ HCT 116 survival (89%) Methanol extract: ↓ MRC‐5 survival (20%), ↓ A549 survival (74%), ↓ HCT 116 survival (81%)	(Cvetković et al. 2025)
Gastroprotective/Gut Microbiota modulation	Commercial dried herb extract (70% ethanol)	In vitro (TIM‐2 colonic model)	20 mL (containing 330 mg phenolic compounds)	↑ growth of *Lactiplantibacillus*, Eggerthellaceae, Erysipelotrichaceae, and Actinomycetota ↑ SCFAs (222.28 mmol), ↑ propionic acid (74.03 mmol), ↑ acetic acid (97.44 mmol), ↑ valeric acid (6.71 mmol)	(Bačić et al. 2025)
Cytotoxic/Pro‐apoptotic	Dichloromethane fraction (AMDF)	In vitro (MDA‐MB‐231 breast cancer cell line)	12.5, 25, 50, and 100 μg/ml	↓ cancer cell viability (IC50: 25 ± 0.08 μg/ml), ↑ *TP53*, ↑ *BAX*, ↑ *CASP9*, ↑ *CASP3*, ↓ cell migration, showed DNA fragmentation	(Qurtam and Nasr 2024)
Renal Protective	Aerial parts extract (70% ethanol)	In vivo (doxorubicin‐induced renal toxicity in rats)	100 and 200 mg/kg	200 mg/kg ↓ serum urea, ↓ uric acid, ↓ creatinine, ↓ inflammation, ↓ NF‐κB, ↓ TNF‐α, ↓ IL‐1β	(Shaiea et al. 2024)
Analgesic/Central Analgesic	Fresh parts (roots, stem, leaves) methanol and chloroform extract	In vivo (acetic acid‐induced writhing test in rats and hot plate test)	100, 200, and 300 mg/kg (given before acetic acid)	300 mg/kg of both extracts ↓ writhing response % inhibition was 58.0% and 62.0% respectively. ↑ Latency time, % inhibition were 59% and 63% after 90 min., respectively.	Adil et al. 2024
Anti‐inflammatory	Fresh parts (roots, stem, leaves) methanol and chloroform extract	In vivo (carrageenan‐induced rat paw edema)	100, 200, and 300 mg/kg	300 mg/kg ↓paw volume, % inhibition were 59.0% and 57.0% respectively	(Adil et al. 2024)
Homeostatic	Flowering aerial parts (hydro‐alcoholic extract)	In vivo (localized bleeding in rat livers)	150 mg/kg	↓ bleeding time, no liver damage or toxicity, no inflammation and granuloms or fibrosis formation	(Bagheri et al. 2024).
Wound healing	AMEO	In vivo (full thickness excisional wounds 2 × 2 cm in rats)	1%, 2%, and 3% w/w ointment	1 and 2% AMEO ↑ wound closure, ↑ hydroxyproline content of tissue, ↑enhanced collagen deposition, ↓ inflammation and ↓ edema	(Ghasemi et al. 2023)
Anti‐inflammatory	Inflorescences and upper dried leaves ethanolic extract YE, and its SAF fractions (YPF, YSF)	In vitro (*H. pylori (*Hp48, Hp53, Hp59 strains) infected AGS cells)	0.08 mg/mL	YE: ↓ IL‐8 (53%–64%) YSF and YPF: ↓ IL‐8 for Hp48 and Hp59 strains YPF: ↓ IL‐8 for Hp53 strain	(Villalva et al. 2022)
Anti‐ulcer/Gastroprotective	Whole plant EO	In vivo (ethanol‐induced gastric ulcer in rats)	100 and 200 mg/kg pre‐treatment	200 mg/kg ↓hemorrhagic injury, ↓ epithelial damage, ↓ edema, ↓ ulcer surface area, ↑ alcian blue binding ability, ↑ gastric pH, ↓ gastric volume, ↑ pepsin activity, ↑ Nrf2 and HO‐1 expressions, ↓ TNF‐α, ↓ IL‐1β, ↓ IL‐6, ↑ PGE_2_, ↑ NO, ↑ Bcl‐2, ↓ Bax, ↓ caspase‐3, ↓ caspase‐9	(Alomair et al. 2022)
Neuroprotective	Flowering branches (aqueous extract)	In vivo (morphine‐induced neurotoxicity in rats hippocampus CA1 region)	100, 250, and 500 mg/kg	500 mg/kg improved spatial memory, ↓neural apoptosis, ↓Bax, ↓caspase‐3 and ↑Bcl‐2, ↓ Bax to Bcl‐2 ratio	(Mozafari et al. 2022)
Anti‐spasmodic	Flower (ethanolic extract)	In vitro (isolated rats uterine contractions, spontaneous and oxytocin‐induced)	0.125, 0.25, 0.5, 1, and 2 mg/ml	2 mg/ml extract significantly ↓spontaneous contractions. 0.5–2 mg/mL ↓amplitude of spontaneous and oxytocin‐induced contractions in dose‐dependent manner	(Eker et al. 2022)
Antidiabetic/Renal Protective	Flower (hydro‐alcoholic extract)	In vivo (diabetic rats)	250 mg/kg	↓kidney weight (1.8 ± 0.12 g), ↓ BUN (7.2 ± 0.68 mg/dL), ↓ creatinine (0.27 ± 0.01 mg/dL), ↓ urea levels (59.8 ± 5.2 mg/dL) regulated apoptotic pathways ↓Bax and ↑Bcl‐2	(Karimi et al. 2021).
Anticancer/Cytotoxic	Flowers (hydro‐alcoholic extract)	In vitro (on AGS gastric cancer cell lines and L‐929 normal fibroblastic cell lines) Three time intervals: 24, 48 and 72 h.	1, 2, 4, 8, 16, 32, 48, 64, and 128 μg/mL	16 μg/mL at 72 h ↓ survival for cancer cells (50%).; ↑ Normal cells survival (85%)	(Hashemi et al. 2021)
Melanogenesis	Compounds isolated from whole plant extract (95% EtOH and 50% EtOH) and its fractions	In vitro (B16 melanoma cells)	1, 10, and 50 μM	At 50 μM Millefoliumins F, G and Austricin ↑ melanin production, ↑ tyrosinase activity of B16 melanoma, activated melanin synthesis	(Li et al. 2021)
Anti‐inflammatory	Millefoliumin A, C, D and austricin (compounds Compounds isolated from whole plant extract (95% EtOH and 50% EtOH) and its fractions)	In vitro (LPS‐induced RAW264.7 cells)	1, 10, and 50 μM	Millefoliumin A and C showed stronger activity inhibited NO production by 50% at 50 μM	(Li et al. 2021)
Antidiabetic	Aerial parts (Hydro‐alcoholic extract)	In vivo (STZ‐induced rats)	100 mg/kg	↑ body weight, ↑ insulin sensitivity, and ↓ blood glucose, ↓ liver enzymes, and ↓ lipid levels; comparable to metformin	(Rezaei et al. 2020).
Anti‐inflammatory	Aerial parts (Aqueous extract)	In vivo (EAE‐induced model for MS)	1, 5, and 10 mg/mouse/day	Delayed behavioral disabilities, prevented weight loss, ↓brain inflammatory cell infiltration, ↓ demyelination, ↑serum TGF‐β (particularly at 10 mg/mouse)	(Vazirinejad et al. 2014)
Gastroprotective	Aerial parts (hydro‐alcoholic extract)	In vivo (ethanol‐ and acetic acid‐induced gastric ulcer in rats)	1, 10, 30, 100, and 300 mg/kg	300 mg/kg ↓ ethanol‐induced gastric lesions (81%) 10 mg/kg ↓ acetic acid‐induced chronic gastric ulcers (65%)	(Potrich et al. 2010)
Anti‐ulcerative	Herb (ethanol extract)	In vivo (human patients with venous leg ulcers)	Ointment containing 7.5% yarrow extract	↓ulcer surface area by 39.64% (44,736 mm^2^ → 27,000 mm^2^)	(Matić et al. 2009)

Abbreviations: *Achillea millefolium* L. dichloromethane fraction; AMEO, *Achillea millefolium* L. essential oil; BAX, Bcl‐2‐associated X protein; Bcl‐2, B‐cell lymphoma 2; BUN, blood urea nitrogen; CASP3, caspase‐3; CASP9, caspase‐9; EAE, experimental autoimmune encephalomyelitis; EO, essential oil; HO‐1, heme oxygenase‐1; IL‐1β, interleukin‐1 beta; IL‐6, interleukin‐6; IL‐8, interleukin‐8; MS, multiple sclerosis; NF‐κB, nuclear factor kappa B; NO, nitric oxide; Nrf2, nuclear factor erythroid 2‐related factor 2; PGE_2_, prostaglandin E2; SAF, supercritical anti‐solvent fractionation; STZ, streptozotocin; TGF‐β, transforming growth factor‐beta; TNF‐α, tumor necrosis factor alpha; TP53, tumor protein p53; YE, yarrow extract; YPF, yarrow precipitated fraction; YSF, yarrow separator fraction.

## 8 Antioxidant Potential

Free radicals are involved in various biochemical processes and can also contribute to the development of diseases such as cancer and inflammatory conditions. In this case, antioxidants protect the body from these free radicals by stopping harmful oxidation (Chandimali et al. 2025). Antioxidants are categorized as enzymatic and non‐enzymatic. Enzymatic antioxidants include primary enzymes (GPx, CAT, and SOD) and secondary enzymes (GR and G6PD). GPx neutralizes peroxides by donating electrons forming selenols and preventing their participation in Fentons's reaction. Catalase (CAT) breaks down hydrogen peroxide (H_2_O_2_) into water and oxygen, while superoxide dismutase (SOD) converts superoxide (O_2_^−^) into H_2_O_2_ for further detoxification by CAT. GR and G6PD help in regenerating glutathione and NADPH, thus sustaining antioxidant activity (Gulcin 2025). Non‐enzymatic antioxidants, such as glutathione, ascorbic acid, carotenoids and flavonoids work by inhibiting oxidative chain reactions. Glutathione acts via GPx and the AsA‐GSH system, while ascorbic acid supports electrons transfer and aids in tocopherol recycling, and carotenoids dissipate excess energy to neutralize oxidative stress (Rao et al. 2025).

The essential oil of *A. millefolium* L. has been reported to possess antioxidant activity. A study compared the antioxidant activities of essential oils from *H. arenarium* and *A. millefolium* L. This study found that *A. millefolium* L. exhibited better scavenging activity in the 2,2‐diphenyl‐1‐picrylhydrazyl (DPPH) assay than *H. arenarium* (7.71 mg/cm3 vs. 8.94 mg/cm3 DPPH radical) (Stanojević et al. 2022). The antioxidant activity of *A. millefolium* L. essential oil was also evaluated using DPPH free radical scavenging and β‐carotene bleaching assays. The study revealed that *A. millefolium* L. oil exhibited strong radical scavenging activity (23.11 ± 0.04 mg/mL), even surpassing Trolox (23.51 ± 0.05 mg/mL). In the β‐carotene bleaching inhibition test, the essential oil (2.05 ± 0.01 μL/mL) also outperformed Trolox (2.06 ± 0.0 μL/mL). The major compounds responsible for this activity were thymol (71%) and carvacrol (16%) (Moghadam 2017). Both studies indicate that *A. millefolium* essential oil exhibits notable antioxidant activity; however, the results differ in magnitude and assay performance. One study reported moderate activity compared to *H. arenarium* , while the other showed strong activity close to or even higher than Trolox in both assays. This difference may likely occur due to differences in the composition of essential oils. Further studies also examined the antioxidant properties of *A. millefolium* L. essential oil using methods such as the DPPH assay, thin layer chromatography‐bioautography (TLC‐bioautography), and preparative thin layer chromatography (PTLC). As a result, thymol exhibited the strongest DPPH radical scavenging activity with the lowest IC50 value of 12.0 ± 0.1 μg/mL. At the same time, bornyl acetate showed the weakest radical scavenging activity with the highest IC50 value of 25 ± 0.1 μg/mL (Moghadam 2017). In one study, the essential oil of *A. millefolium* L. also demonstrated the highest antioxidant activity using the ABTS (2,2′‐azino‐bis(3‐ethylbenzothiazoline‐6‐sulfonic acid)) method (0.34 mg/mL), while showing the lowest activity in the DPPH and Fe‐reducing methods (IC50: 32.75 mg/mL, RP50: 127.87 mg/mL) (Vidic et al. 2016).

Another study investigated the impact of different extraction solvents on TPC and TFC, as well as the antioxidant activity of *A. millefolium* L. flowers in Kosovo. It was found that methanolic extracts had the highest phenolic content (52.6 ± 0.3 mg of GAE/g), flavonoid content (27.8 ± 0.2 mg of CE/g), and DPPH radical scavenging activity (51.9 ± 0.3 μMol/g). In comparison, aqueous extract had the lowest total phenolic content (18.2 ± 0.2 mg GAE/g), the lowest total flavonoid content (5.2 ± 0.2 mg CE/g), and the lowest DPPH scavenging activity (24.3 ± 0.4 μMol/g) (Koraqi et al. 2022). Other studies have also evaluated the antioxidant activity of *A. millefolium* L. leaf and stem extracts using the DPPH and nitric oxide assays. They found that the leaf hydro‐alcoholic extract exhibited better DPPH scavenging activity (14.80%) than the stem extract (12.30%). In contrast, the inhibition shown by the leaf extract (12.88%) was also higher than that of the stem extract (10.90%) in the nitric oxide assay (Yaseen et al. 2017). The antioxidant activity of *A. millefolium* L. flowers (methanol from fresh and dried material, ethyl acetate and water extracts from fresh flower) was assessed using DPPH assay at 25–175 μg/mL and compared with Trolox. As a result, methanol extract from dried material exhibited the highest activity, with 19.2% radical scavenging at 175 μg/mL (6.8 μg/mL Trolox equivalent). In contrast, the ethyl acetate extract showed the lowest activity with 6.17% scavenging at 175 μg/mL (2.2 μg/mL Trolox equivalent) (Paduch et al. 2008). Furthermore, one study also compared the free radical scavenging activity and total flavonoid content in ethanol extracts of the aerial parts of six different *Achillea* species, including *A. millefolium* L. They found that *A. micrantha* had the highest DPPH radical scavenging activity (IC50 value: 32.92 μg/mL), while *A. millefolium* L. exhibited moderate activity (IC50 value: 49.93 μg/mL). The highest flavonoid content was observed in *A. vermicularis* (58.17 ± 0.82 μg/mg), followed by *A. millefolium* L. (42.46 ± 1.46 μg/mg) (Nickavar et al. 2006). Table 3 highlights the antioxidant activities of *A. millefolium* L. extracts from different plant parts, evaluated using various assays.

**TABLE 3:** Antioxidant activity of *A. millefolium* L.

Location	Parts and forms	Assay (s)	Antioxidant activity	References
Poland	Aerial parts extract (hydro‐ethanolic made by UAE and SFE method)	FRAP, DPPH	Higher activity in extract obtained by UAE method (DPPH: 70.00% ± 0.14%; FRAP: 1.53 ± 0.03 mmol TE/L)	(Michalak et al. 2025)
Turkiye	Methanol‐chloroform extract of plant (later dissolved in methanol/50% aqueous methanol)	DPPH, CUPRAC	CUPRAC: 32.78 ± 0.641% DPPH: 8.61% ± 0.211%	(Baran et al. 2025)
Poland	In vitro microshoots (AmIV), herbs (AmH) and leaves (AmL) from field‐grown plant (aqueous, 50% EtOH, 96% EtOH extracts)	DPPH	Highest activity in 50% EtOH extracts of AmIV (EC50: 400.05 ± 45.47 μg/mL), AmH (EC50: 8.22 ± 2.32 μg/mL) and AmL (EC50: 5.10 ± 0.04 μg/mL)	(Czech et al. 2023)
India	Flower oil	DPPH	Max: 85.58% at 300 μg/mL; Min: 40% at 50 μg/ml (Min.)	(Nazir et al. 2022)
Switzerland/Germany	Aerial parts extracts (dichloromethane, n‐butanol, ethyl acetate)	DPPH, Folin–Ciocalteu method	ethyl acetate showed higher activity (IC50: 17.0 ± 0.3 μg/mL), TPC: ethyl acetate fraction 175 ± 1.0 mg GAE/g DW	(Salomon et al. 2021)
Iran	Flower and leaf EO (flowering stage)	DPPH	Higher in flowers (0.91 mg/mL) vs. leaves (0.94 mg/mL)	(Ahmadi‐Dastgerdi et al. 2017)
Iran	Aerial parts EO (stem, flowers and leaves)	GC/MS, DPPH, TLC‐bioautography, and PTLC	Thymol: (IC50: 12.0 ± 0.1 μg/mL, strongest activity) Carvacrol: (IC50: 13.43–14.43 μg/mL, second strongest activity) (↓ IC50 indicates ↑ DPPH radical scavenging activity)	(Kazemi 2015a, 2015b)
Iran	Aerial parts EO	DPPH, FRAP, β‐Carotene bleaching, TPC	TPC: 531 ± 1.34 GAE/g (Highest activity) DPPH: 22.11 ± 0.06 mg/mL FRAP: 360.1 ± 0.12 μmol Fe^2+^/g EO; β‐carotene inhibition: 1.1 ± 0.12 μL/mL	(Kazemi 2015a, 2015b)
Turkey	Herbal parts (water‐soluble methanolic extract), Aerial part EO	DPPH, Superoxide radicals inhibition, Hydroxyl radical scavenging, Lipid peroxide inhibition	Highest activity of EO in DPPH (IC50: 1.56 ± 0.03 μg/mL), Hydroxyl scavenging (2.70 ± 0.03 μg/mL) and Lipid peroxidation (13.50 ± 0.07 μg/mL) Highest activity of extract in Superoxide inhibition (304.00 ± 5.10 μg/mL)	(Candan et al. 2003)

Abbreviations: CUPRAC, cupric reducing antioxidant capacity; DPPH, 1,1‐diphenyl‐2‐picrylhydrazyl; EO, essential oil; FRAP, ferric reducing antioxidant power; GC/MS, gas chromatography/mass spectrometry; PTLC, preparative thin layer chromatography; SFE, supercritical fluid extraction; TLC, thin‐layer chromatography; TPC, total phenolic content; UAE, ultrasound‐assisted extraction.

In addition to chemical assays, *A. millefolium* L. extracts have also been shown to modulate antioxidant enzyme activity in in vivo models. In one study, 100 and 200 mg/kg of *A. millefolium* L. extract (70% ethanol) of aerial parts significantly improved SOD, CAT, GSH, GPx and TAC activities in the kidney while reducing MDA and NOx levels in doxorubicin‐induced renal toxicity in rats. Stronger effects were observed in the 200 mg/kg group (*p* < 0.05–0.001) (Shaiea et al. 2024). Another study also investigated the effect of *A. millefolium* hydro‐alcoholic extract on cisplatin‐induced ocular toxicity and found that a 400 mg/kg dose increased SOD activity (approximately 5 ng/mL), improved antioxidant activity (approximately 1.4 mmol Trolox Equiv./L), and decreased MDA (< 250 ng/mL) (Okkay et al. 2021). Moreover, in rat models, the hydro‐alcoholic extract of *A. millefolium* L. aerial parts (300 mg/kg) improved glutathione (GSH) levels (1187 ± 85 μg GSH/g tissue) and superoxide dismutase (SOD) activity (2.5 ± 0.1 U SOD/g tissue) in ethanol‐induced ulcers, while 10 mg/kg of extract also improved GSH (1597 ± 177 μg GSH/g tissue) and SOD activity (2.9 ± 0.2 U SOD/g tissue) in acetic acid‐induced ulcers (Potrich et al. 2010). Overall, the antioxidant activity of *A. millefolium* L. is demonstrated through both direct radical scavenging assays (DPPH, ABTS, β‐carotene) and enhancement of antioxidant enzymes (SOD, GSH, GPx, CAT). However, the reported results show variation across different assays, extract types, and plant parts. Moreover, most studies are based on chemical assays rather than biological assays, and the actual in vivo relevance remains unclear. These limitations highlight the need for well‐designed clinical studies to further verify and confirm its antioxidant potential.

## 9 Gastroprotective Role

The gastrointestinal tract is a vital organ of the human body and is particularly susceptible to various parasitic and infectious diseases. However, herbal plants have been used to treat these disorders because of their effectiveness, fewer side effects, and potential in drug development (Sulaiman et al. 2022). *A. millefolium* L. is also one of the medicinal plants that are rich in bioactive components like essential oils, flavonoids, tannins, sesquiterpene lactones, etc., and is used for the treatment of abdominal pain, diarrhea, constipation, gastroenteritis, stomach ulcer, and other issues (Bahmani, Abdi, et al. 2014; Bahmani, Zargaran, and Rafieian‐Kopaei 2014).

Several studies have reported the gastroprotective properties of *A. millefolium* L. and according to one such study, it was found that the combination of *G. glabra* (110 mg), *M. chamomilla* (55 mg) and *A. millefolium* L. (165 mg) reduced constipation in 60.8%, diarrhea in 80.4% and mucus secretion in 88.2% of patients with irritable bowel syndrome after 2 weeks of intervention (Rahimi et al. 2023). Another study that reports the gastroprotective effect of poly‐herbal combination shows that the herbal mixture of *B. carterii*, *Z. officinale* , and *A. millefolium* L. significantly reduced the scores of abdominal pain severity (32.29 ± 17.56), abdominal pain frequency (4.25 ± 2.84), bloating (40.20 ± 224.20), anxiety (16.5 ± 3.40), and depression (14.41 ± 4.16) in patients having irritable bowel syndrome after 1 month of intervention (Kazemian et al. 2017). Both studies show that *A. millefolium* L. based poly‐herbal formulations improve gastrointestinal symptoms in irritable bowel syndrome; however, outcomes differ in measured parameters and magnitude of improvement. Rahimi et al. (2023) observed stronger effects on specific symptoms such as diarrhea, constipation, and mucus secretion in a short term period; whereas Kazemian et al. (2017) reported comparatively moderate improvements in pain, bloating, and psychological parameters over a longer duration. These differences may be due to variations in herbal combinations, plant composition, treatment time, and assessed parameters.

In another study, Bais et al. (2014) found that doses of 300 and 450 mg/kg of the methanolic extract of *A. millefolium* L. leaves significantly lowered gastrointestinal parameters in castor oil‐induced diarrheal rats. The distance traveled by the charcoal meal was reduced to 25 ± 1.679 cm and 17 ± 2.534 cm, respectively. Similarly, total stool frequency decreased to 3.6 ± 0.5099 min and 2.9 ± 0.4427 min, and the frequency of wet stools dropped by 1.01 ± 0.74 min and 0.96 ± 0.54 min, respectively. Abdi et al. (2016) found that in patients suffering from gastroenteritis, 0.5 mL/kg *A. millefolium* L. distillate administered every 8 h, in addition to the usual treatment, reduced recovery time in patients. In the control group, the duration of recovery was 1.86 ± 0.71 days, while in the intervention group it was 1.31 ± 0.71 days. It was also found that *A. millefolium* L. distillate had no toxic side effects. Collectively, studies indicate that *A. millefolium* L. shows gastroprotective and antidiarrheal effects through reduction of gastric lesions and modulation of intestinal motility. However, most of the evidence originates from pre‐clinical studies or poly‐herbal formulations, making it difficult to determine the direct contribution of this plant. Variations in extract types, dosages, and experimental protocols further limit comparability. Therefore, well‐designed clinical studies are needed to validate its efficacy and applicability.

## 10 Antidiabetic Potential

Diabetes mellitus is characterized as a chronic metabolic disease in which the blood glucose level is increased above normal due to insulin deficiency or increased resistance. Herbal plants are incorporated into the management of diabetes, as bioactive compounds such as anthocyanins, alkaloids, flavonoids, and phenols help regulate blood sugar levels through various mechanisms (Noman, Sultan, Mazhar, et al. 2025; Noman, Sultan, Maaz, et al. 2025). *A. millefolium* L. is found to possess antidiabetic activity because of various modes of interaction and mechanisms. One such mechanism is the inhibition of α‐ glucosidases enzymes. These enzymes are involved in the absorption of carbohydrates. Still, since *A. millefolium* L. inhibits their activity, the hyperglycemic peak is reduced, and as a result, the risk of diabetes is alleviated. This activity of *A. millefolium* L. can be attributed to the presence of flavonoids, such as luteolin, and others. Another mechanism is that *A. millefolium* L. increases insulin secretion and cytosolic calcium levels, which help regulate blood glucose levels. Lastly, *A. millefolium* L. also activates a receptor called PPARγ, also known as Peroxisome proliferator‐activated receptor gamma, which is responsible for regulating insulin sensitivity and glucose metabolism. The activation of PPARγ increases GLUT4 expression in the body, which is also involved in the uptake of glucose into the muscles and tissues, resulting in reduced blood glucose levels. The *A. millefolium* L. and its extracts may also inhibit enzymes like PTP1B and 11βHSD1, which enhance insulin sensitivity. Inflammatory markers, such as interleukin‐1β (IL‐1β) and inducible nitric oxide synthase (iNOS), contribute to insulin resistance. *A. millefolium* L. has also been shown to reduce the expression of these inflammatory cytokines (Chávez‐Silva et al. 2018).

A randomized clinical trial in Iran evaluated the effects of oral supplementation of *A. millefolium* L. aqueous extract capsules (500 mg/day) prepared from flowering aerial parts in 60 patients with type 2 diabetes for 3 months. The results showed a non‐significant reduction in HbA1C levels in the intervention group (baseline: 8.61 ± 1.61; post‐intervention: 8.35 ± 1.86). However, the neuropathic score was reduced significantly in the plant‐treated group (4.61 ± 2.07) compared to the placebo group (6.30 ± 1.44), where placebo capsules contained 500 mg cellulose (Basirat et al. 2025). In another study, *A. millefolium* L. aqueous extract (flowering branches) at the doses of 300 and 600 mg/kg reduced serum blood glucose (285.3 ± 23.25 mg/dL and 284.0 ± 19.98 mg/dL) and increased body weight (229.2 ± 5.712 g and 237.2 ± 4.956 g) in STZ‐induced diabetic neuropathic rats. These doses also elevated the nociception threshold, exhibited antiapoptotic and anti‐inflammatory effects by decreasing cytochrome‐c, Bax/Bcl2 ratio, cleaved caspase‐3, TNF‐α and NF‐κB expressions in lumbar spinal cord tissue of diabetic rats. The 600 mg/kg extract significantly increased Bcl2 and decreased Bax expression (Moradi et al. 2024). Both studies suggest beneficial effects in diabetes‐related conditions, but results differ between clinical and animal models. The clinical study reported limited glycemic improvement although neuropathic symptoms improved significantly. The animal model, on the other hand, reported a clear reduction in blood glucose levels, along with anti‐inflammatory and anti‐apoptic effects. These differences may be attributed to variation in experimental models, dosage, and physiological response.

The methanolic extracts of aerial parts of five *Achillea* species were evaluated for their antidiabetic and anti‐inflammatory activity using the α‐glucosidase inhibition and anti 5‐lipoxygenase activity. Among all tested species, *A. nobilis* subsp. *neilreichii* and *A. crithmifolia* showed the strongest inhibitory effect against the α‐glucosidase enzyme (IC50: 245.50 and 298.60 μg/mL, respectively). As for the anti‐inflammatory effect, *A. crithmifolia* was more effective than *A. nobilis* subsp. *neilreichii* (IC50: 90.30 and 99.06 μg/mL, respectively). In contrast, *A. millefolium* L. extract exhibited the weakest activity among all species with IC50 values of 1201 μg/mL (α‐glucosidase inhibition) and 471.60 μg/mL (anti‐5‐LOX) (Gürbüzkol and Bitiş 2025). In another study, aqueous extracts of *A. millefolium* L. (100 mg/kg) and *T. polium* (100 mg/kg) as well as their combined extract reduced fasting blood sugar (FBS) (373 ± 179 mg/dL; 271 ± 125 mg/dL; 363 ± 188 mg/dL), cholesterol and triglycerides up to 40 mg/dL, and LDL below 20 mg/dL in STZ‐induced diabetic rats. This effect may be attributed to the flavonoids and phenolic acids present in both plants, as these compounds scavenge free radicals and possess antioxidant activity. Additionally, these plants exhibit antidiabetic effects by inhibiting the expression of Nuclear Factor Kappa B (NF‐κB), cyclooxygenase‐2 genes (COX‐2), reducing pro‐inflammatory cytokines, protecting β cells from apoptosis, and improving carbohydrate metabolism in the liver (Mir et al. 2018).

**FIGURE 4:**
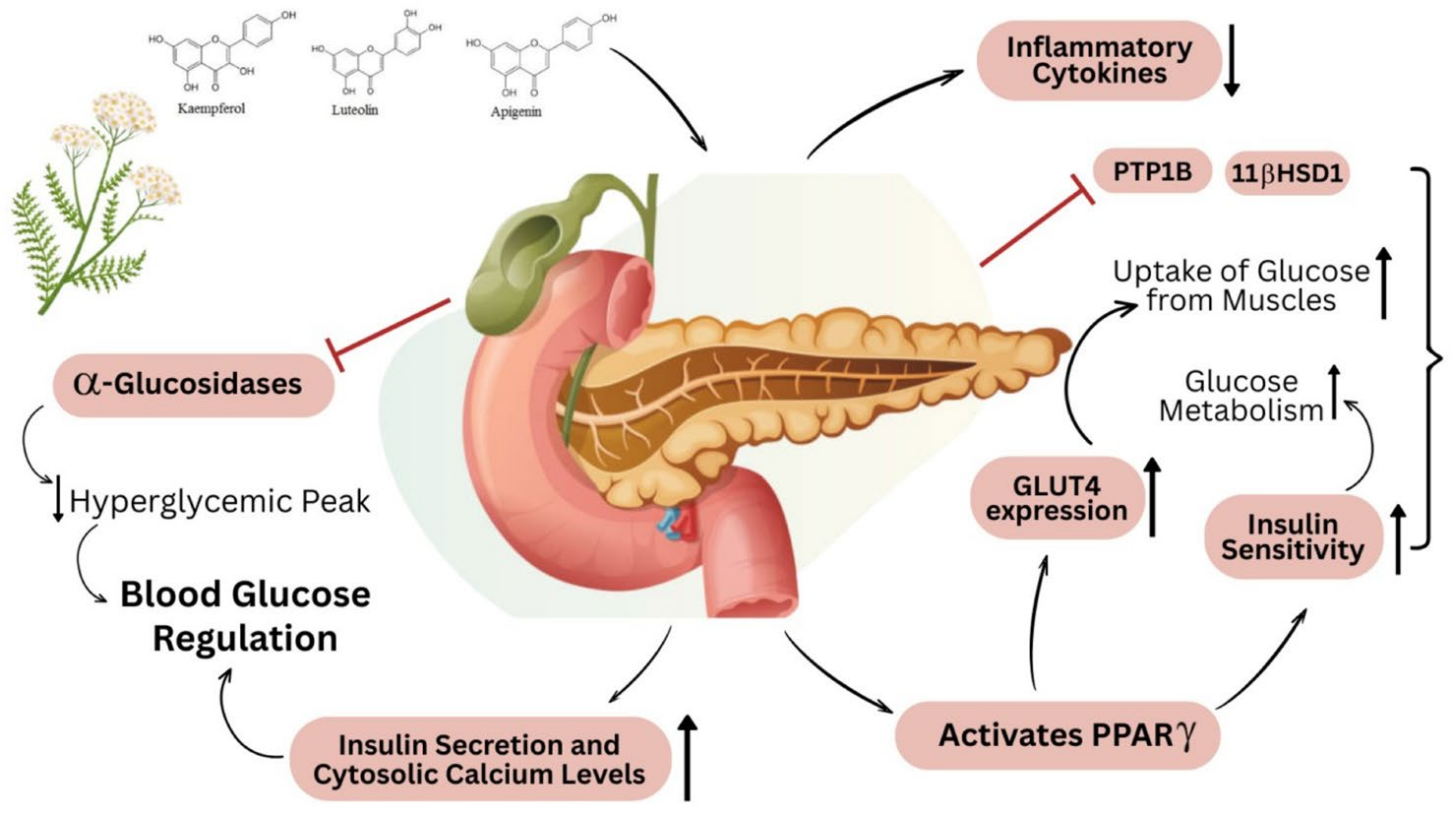
Antidiabetic potential of *Achillea millefolium* L. via downregulating inflammatory cytokines, inhibiting α‐glucosidases, PTP1B, and 11βHSD1, improving insulin sensitivity, GLUT4 expression, insulin secretion, cytosolic calcium level, and glucose uptake, and activating PPAR‐ γ.

Additionally, 90 and 270 mg/kg of *A. millefolium* L. aqueous extract decreased blood glucose, creatinine, and urea levels, and improved RBC (red blood cells), WBC (white blood cells), and platelet parameters, as well as renal tissue health, in diabetic rats compared to controls (Zangeneh 2018). *A. millefolium* L. exerts its effect by downregulating the expression of IL‐1β and iNOS and protecting β cells in the pancreas. Zolghadri et al. (2014) reported that *A. millefolium* L. hydro‐alcoholic extract (100 mg/kg/day) inhibited *IL‐1β* and *iNOS* gene expression in STZ‐induced diabetic rats by 56% and 55%, respectively, improved insulin levels (15.72 ± 0.56μU/mL), and reduced glucose levels (187.17 ± 12.02 mg/dL). Moreover, the extract also reduced the IL‐1β/GAPDH mRNA ratio and the iNOS/GAPDH mRNA ratio to 1.92‐ and 1.76‐fold, respectively. Mustafa et al. (2012) found that the methanolic and aqueous extract of *A. millefolium* L. reduced glucose level by 50.70% and 44.25% in diabetic rats and also decreased TGL (triglycerides) (95.50 ± 6.1 mg/dL and 111.66 ± 7.9 mg/dL), VLDL (very low‐density lipoprotein) (47.83 ± 1.9 mg/dL and 39.66 ± 1.5 mg/dL), cholesterol (138.50 ± 6.5 mg/dL and 144.83 ± 4.9 mg/dL), SGOT, SGPT, and ALP at the dose of 500 mg/kg body weight. The extracts were found to be protective against the cytotoxic effects of alloxan monohydrate on pancreatic beta cells. Overall, *A. millefolium* L. shows antidiabetic activity through diverse pathways, including modulation of carbohydrates absorption, activation of PPARγ and GLUT‐mediated glucose uptake, and suppression of pro‐inflammatory markers like IL‐1β and iNOS. These findings support its potential in regulating blood glucose level. However, most studies are based on animal models, and variations in extract preparation, dosing, and study protocols highlight the need for controlled clinical investigations to confirm its therapeutic efficacy and safety in humans. Figure 4 displays the antidiabetic potential of *A. millefolium* L.

## 11 Anti‐Spasmodic Effect

Gastrointestinal disorders such as irritable bowel syndrome, colic, and others are common complications that cause painful smooth muscle spasms. Although synthetic anti‐spasmodic drugs are used to relieve this pain, their increasing side effects have raised an interest in medicinal plants, as these plants offer much safer alternatives to manage these disorders (Rauf et al. 2021). *A. millefolium* L. also possesses anti‐spasmodic activity by reducing smooth muscle contraction in both gastrointestinal and uterine muscles. This effect is attributed to its flavonoid derivatives, such as luteolin, apigenin, and quercetin, which exhibit antagonistic effects on calcium release in smooth muscles (Yaeesh et al. 2006). Additionally, their vasodilating properties also contribute to reducing these contractions (Yadegari et al. 2015).

Several studies have demonstrated the anti‐spasmodic activity of *A. millefolium* L. A study found that the combination of the hydro‐alcoholic extract (1%) with potassium chloride (KCl, 60 mM) or acetylcholine (1 μg/mL) significantly reduced ileum contraction. The mean percentage of ileum contraction relief in Wistar rats who received extract and KCl ranged from 62.96% ± 11.08% to 59.96% ± 11.8%, and in rats who received extract and acetylcholine, it ranged from 53.16% ± 12.06% to 54.16% ± 12.06%, respectively, which was significantly lower as compared to the groups only treated with KCl or acetylcholine alone (18.83% ± 4.91% and 18.31% ± 11.12%, respectively), representing that the extract can relieve ileum contractions (Sedighi et al. 2013). The analgesic, anti‐inflammatory, and anti‐spasmodic activities of hydro‐alcoholic extracts of *A. millefolium* L. and *A. vulgaris* were investigated. It was found that 500 and 1000 mg/kg of *A. millefolium* L. significantly inhibited abdominal contortions by 65% and 23%, respectively. Still, it did not affect intestinal transit, hot plate response times, or formalin test responses (Pires et al. 2009). Hydro‐alcoholic extract significantly reduced contractions induced by KCl and acetylcholine, with no effect from propranolol or L‐NAME (Nω‐nitro‐L‐arginine methylester hydrochloride) on isolated ileum contractions in rats. According to a study, the anti‐spasmodic effect of *A. millefolium* L. may occur due to the blockade of voltage‐dependent calcium channels, with the potential involvement of nitric oxide and adrenergic systems (Moradi et al. 2013). In summary, *A. millefolium* L. demonstrates anti‐spasmodic and muscle‐relaxing effects through flavonoid‐mediated inhibition of calcium channels, modulation of acetylcholine and potassium induced contractions, and regulation of vascular tone. While pre‐clinical evidence shows promising results, standardized clinical trials and validations are needed to confirm the plant's therapeutic potential.

## 12 Anti‐Ulcerative and Renal‐Protective Impact

Ulcers are swollen lesions in the mucosal lining of the gastrointestinal tract caused by an imbalance between mucosal defenses and harmful factors. Several medicinal plants have shown dose‐dependent ulcer prevention in studies with low toxicity. Their protective effects are linked to bioactive compounds, such as terpenoids, flavonoids, and tannins, which can enhance mucosal defense and promote healing (Gadekar et al. 2010). *A. millefolium* L. also contains compounds such as borneol and camphor (terpenoids), apigenin, and luteolin (flavonoids) that exhibit wound healing and antioxidant activities (Saadat et al. 2024; Ali et al. 2017).

A study has demonstrated the anti‐colitis activity of the hydro‐alcoholic and essential oils of *A. millefolium* L. in a murine colitis model. Results showed that administration of 400 and 600 mg/kg/day of extract and 125 and 250 μL/kg/day of EO improved markers like ulcer index, total colitis index (9.0 (4‐13); 4.5 (1‐9); 6.0 (3‐7); 4.5 (2‐8) respectively), colon weight/length ratio, weight gain in rats (177.0 ± 3.8, 204.0 ± 8.4, 191.3 ± 5.6, 186.0 ± 4.0 g respectively) while MDA levels and myeloperoxidase (MPO) activity were decreased in the case group as compared to the control group (*p* < 0.05). These therapeutic effects are linked to its mode of action as *A. millefolium* L. helps in scavenging free radicals, minimizing acid secretion, and enhancing mucosal protection. It also promotes wound healing and cell proliferation in the stomach, and reduces spasms in the ileum and colon to ease pain. Moreover, it also modulates inflammatory markers by decreasing the expression of TNF‐α, NO, interleukin‐6 (IL‐6), and increasing the expression of interleukin‐10 (IL‐10) (Hadavi‐Siahboomi et al. 2022).

Beyond its wound‐healing properties, *A. millefolium* L. has also demonstrated renal‐protective effects, which highlights its therapeutic potential and versatility. Nephrolithiasis (renal stones) is a common urinary tract issue that lacks a definite treatment. Herbal plants and remedies have shown promise in managing this condition, as they help modify urinary ion balance and inhibit crystallization (Al‐Mamoori et al. 2019). *A. millefolium* L. can also prevent and help in dissolving calcium oxalate (CaOx) by minimizing urinary oxalate levels and maximizing urinary citrate levels, which inhibits crystal formation and, in turn, increases urine output because of its diuretic effects. Moreover, its anti‐inflammatory, antioxidant, and antimicrobial properties help protect renal epithelial cells from damage and potential infections that can cause kidney stones. According to a study, the hydro‐alcoholic extract of *A. millefolium* L. given from day 1 and day 15 of the experiment (200 and 400 mg/kg extract) reduced urinary oxalate concentration (1.44 ± 0.04 mg/dL and 1.26 ± 0.03 mg/dL) and CaOx deposition while increasing urinary citrate (14.6 ± 2.3 mg/dL and 15.49 ± 2.01 mg/dL) concentration in rats with ethylene glycol‐induced nephrolithiasis (Bafrani et al. 2014). *A. millefolium* L. also plays a protective role in managing chronic kidney disease (CKD) by decreasing plasma nitrite and nitrate levels. According to a study by Vahid et al. (2012), administering 1.5 g of powdered *A. millefolium* L. flower 3 days a week for 2 months reduced plasma nitrite (0.82 ± 0.51 μmol/L to 0.63 ± 0.42 μmol/L) and nitrate concentrations (50.55 ± 17.92 μmol/L to 44.09 ± 17.49 μmol/L) in patients with CKD. From these studies, we have concluded that *A. millefolium* L. exhibits anti‐ulcerative, wound healing and renal‐protective effects via antioxidant activity, modulation of inflammatory cytokines (TNF‐α, IL‐6, IL‐10), enhancement of mucosal integrity and regulation of urinary ions to prevent calcium oxalate deposition. These combined actions support tissue repair, ulcer reduction and protection against renal complications. However, most evidence comes from animal models and small‐scale human studies with short term outcomes. Further research is needed to confirm long term clinical safety and efficacy in large populations.

## 13 Anticancer Activity

Cancer is becoming a global health challenge, with increasing incidence and rising mortality rates each year. Cancer development is influenced by multiple factors including poor hygiene in diet, pathogenic infections, and a polluted environment. It is associated with processes such as inflammation, oxidative imbalance, DNA damage and genetic alterations (Noman, Sultan, Mazhar, et al. 2025; Noman, Sultan, Maaz, et al. 2025). Although conventional therapies, such as drugs and radiotherapy, are commonly used, herbal plants and their bioactive compounds have also shown potent anticancer activities. Many medicinal plants exhibit cytotoxic effects and enhance the immune response. Phytochemicals present in these plants can improve treatment efficacy by modulating molecular pathways, enhancing antioxidant activity, inducing cell cycle arrest, and promoting apoptosis (Maaz et al. 2025). *A. millefolium* L. contains bioactive components like caffeic acid, camphene, and kaempferol that are responsible for its anti‐inflammatory and antitumor activities. Caffeic acid inhibits nitric oxide (NO) production, as well as NF‐κB‐mediated and UVB‐mediated COX‐2 production, and suppresses COX‐2 and iNOS activity. Camphene promotes cell death in abnormal cancer cells. At the same time, kaempferol enhances the production of activated macrophages and modulates cytokine expression by downregulating lipopolysaccharide‐induced tumor necrosis factor alpha (TNF‐α) production (Farasati Far et al. 2023). Similarly, its hydro‐ethanolic extract contains high concentrations of chlorogenic acid derivatives, which are known for their ability to inhibit cancer cell growth (Pereira et al. 2018). These derivatives disrupt mitogen‐activated protein kinase (MAPK) and phosphatidylinositol 3‐kinase/protein kinase B (PI3K/AKT) signaling pathways, inactivate NF‐kB, activator protein 1 (AP‐1), and signal transducer and activator of transcription 3 (STAT3), thereby hindering cancer cell proliferation (Tsai et al. 2013).

A study has evaluated the cytotoxic, phytotoxic, and insecticidal activities of methanolic, aqueous, and chloroform extracts of *A. millefolium* L. and *C. villosum* . For *A. millefolium* L., it was found that the methanolic extract of *A. millefolium* L. (1000 μg/mL) showed high cytotoxic activity on brine shrimps (LD50: 52.60 μg/mL), phytotoxic effects (FI50: 6.60 μg/mL) on *L. minor* , and insecticidal toxicity against *S. dadkhani* (LD50: 4.17 μg/mL) and *T. granarium* (LD50: 242.22 μg/mL) while the chloroform extract exhibited the highest insecticidal activity against *Tribolium castaneum* (LD50: 31.41 μg/mL) (Adil et al. 2024).

*A. millefolium* L. methanolic extract combined with bleomycin reduced cell viability (60% ± 6% and 49% ± 6%) in prostate cancer (DU‐145) cells at higher concentrations (1000 and 2000 μg/mL), but it did not cause any toxicity in non‐malignant fibroblast (HFFF2) cells (Shahani, Rostamnezhad, et al. 2015; Shahani, Hamzekanlu, et al. 2015). Another study also determined the anti‐proliferative activity of aqueous, ethanolic, and methanolic extracts of *A. millefolium* L. flowers and leaves on the MCF‐7 breast cancer cell line. The results showed a significant difference in cell viability between aqueous and hydro‐alcoholic extracts of leaf and flower, and ethanolic and methanolic extracts of flower, while no significant difference was noted in methanolic and ethanolic extracts of leaf. Among all extracts, the ethanolic extract of flowers showed the highest cytotoxic activity (11.77 μg/mL and 7.3 μg/mL at 24 and 48 h, respectively) (Amini Navaie et al. 2015). Both studies show that *A. millefolium* L. exhibits anticancer related effects across different cell lines. Shahani, Rostamnezhad, et al. (2015); Shahani, Hamzekanlu, et al. (2015) reported selected cytotoxicity in cancer cells, while sparing normal fibroblasts, indicating potential safety. In contrast, Amini Navaie et al. (2015) reported stronger anti‐proliferative effects of flowers' ethanolic extract as compared to other extracts. The difference in results may occur due to variations in plant parts, extract types, exposure time, dosages, or cell models used.

**FIGURE 5:**
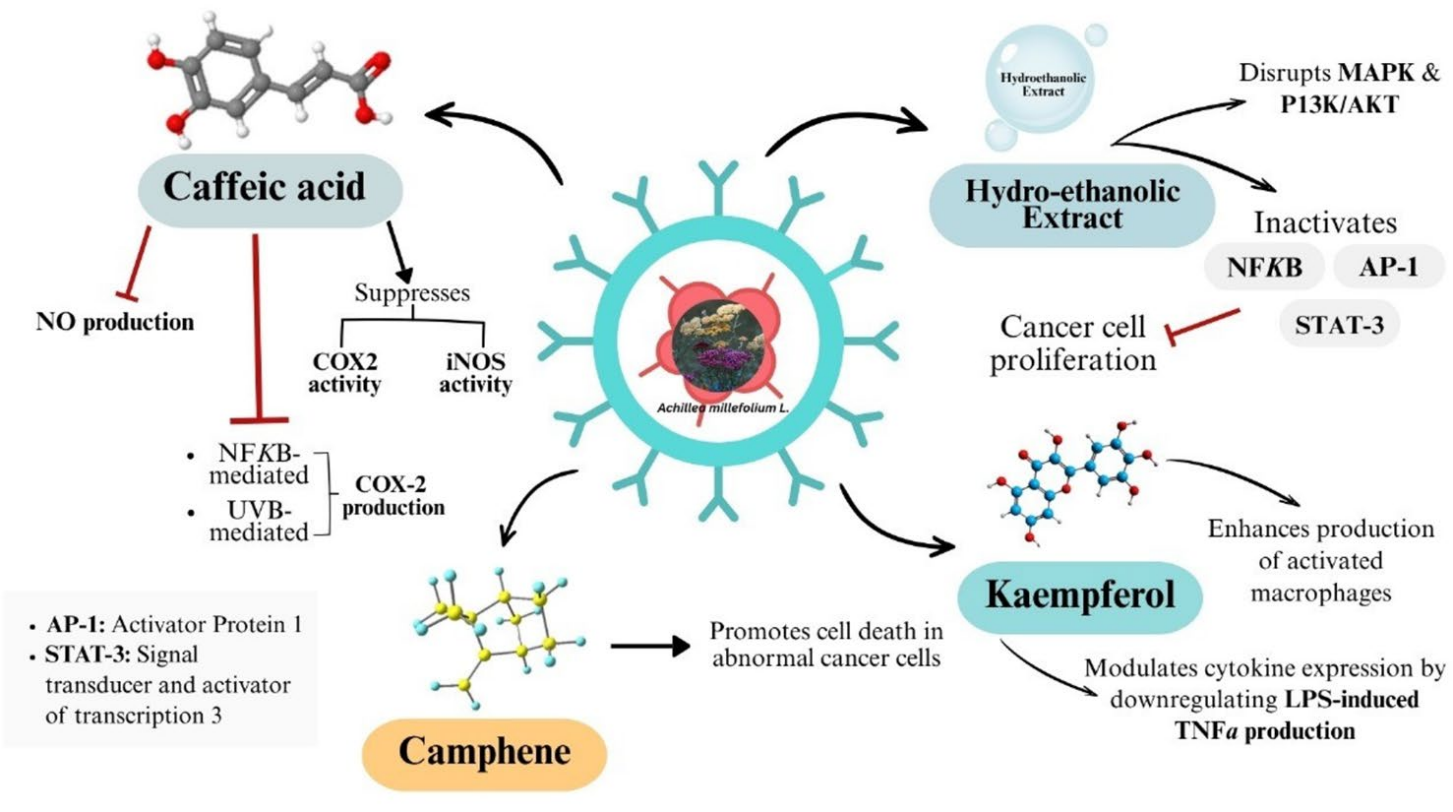
Anticancer activity of *Achillea millefolium* L. through downregulating TNF‐α, inhibiting cell proliferation and NO production, apoptosis induction, suppressing COX‐2 and iNOS, inactivating STAT3, AP‐1, and NFκ‐B, and disrupting MAPK and PI3K/AKT.

Methanolic extract of *A. millefolium* L. was found to be radio‐protective against genotoxicity induced by ionizing radiation in human lymphocytes, as it reduced micronuclei frequency in extract‐treated lymphocytes, particularly at the level 200 μg/mL (Shahani, Rostamnezhad, et al. 2015; Shahani, Hamzekanlu, et al. 2015). Another study also revealed the in vitro anti‐proliferative effects of n‐hexane, chloroform, aqueous methanol, and aqueous extracts on three human tumor cell lines (HeLa, MCF‐7, and A431). The results showed that the chloroform extract (10 μg/mL) exhibited the highest inhibition on HeLa and MCF‐7 cells among the other extracts. In contrast, compounds identified through bioactivity‐guided fractionation showed that a flavonoid named centaureidin exhibited the strongest anti‐proliferative activity against these cancer cell lines (IC50 of 0.0819 μM for HeLa and 0.1250 μM for MCF‐7). Other compounds like casticin and paulitin showed effective inhibition (IC50: 1.286–4.76 μM) while luteolin and isopaulitin showed moderate effects (IC50 6.95–32.88 μM) on all cell lines (Csupor‐Löffler et al. 2009). Furthermore, Düsman et al. (2013) found that aqueous extracts of *A. millefolium* L. (35 g/L) and *B. forficata* (4.65 g/L) significantly inhibited chromosomal alterations, with *A. millefolium* L. reducing alterations by 68%–71% in pre‐treatment and 67% in post‐treatment against cyclophosphamide‐induced mutations in Wistar rat bone marrow cells. Huo et al. (2013) determined 9 flavonoid compounds from the flowers of *A. millefolium* L., such as artemetin, casticin (vitexicarpin), chrysoeriol, jaceidin, centaureidin, apigenin, quercetagetin 3,3′‐dimethyl ether, luteolin, and 8,8′‐bi‐3‐O‐methylquercetin. 8,8′‐bi‐3‐O‐methylquercetin was a new compound found. Regarding their anti‐proliferative activity, casticin, centaureidin, and quercetagetin 3,3′‐dimethyl ether were active against the breast cancer cell line MCF7WT, with IC50 values of 0.59, 0.91, and 1.19 μM, respectively. While only casticin and centaureidin were active against the prostatic cell line PC‐3 (IC50: 1.24 and 1.74 μM). In contrast, *A. millefolium* L. leaves extract (50% ethanol) showed strong cytotoxic effects in vitro assays on porcine intestinal epithelial cells (IPEC‐1) at all tested doses (125, 250, 500 and 1000 μg/mL). It caused the loss in cell adherence (28.34%, 17.61%, 14.41%, and 15.72%) while an increase in metabolic activity was observed (46.03%, 50.45%, 59.51% and 76.47%), likely due to its polyphenolic content (Sowa et al. 2020). Similarly, aqueous extract of *A. millefolium* L. fresh leaves at 10 and 20 mg/mL also showed cytotoxic effects on root cells of lettuce ( *Lactuca sativa* ) as it caused reduction in the mitotic index (*p* < 0.05) while 30 mg/mL of extract stopped seed germination completely. The extract also induced chromosome abnormalities such as breaks, bridges, stickiness, and micronuclei (*p* < 0.05) (Sousa and Viccini 2011). In another study, the genotoxic effects of *A. millefolium* L. flowers essential oil (0.13, 0.19 and 0.25 μL/mL) was assessed using *Aspergillus nidulans* diaploid strain (A757//UT448). As a result, essential oil at the concentrations of 0.19 and 0.25 μL/mL elevated the numbers of yellow and white mitotic recombinants with mitotic segregation index (MSI) values of 2.0 and 3.7, respectively (Sant'Anna et al. 2009). All in all, *A. millefolium* L. shows considerable anticancer potential by reducing cancer cell viability and proliferation, inducing apoptosis, modulating key signaling pathways (MAPK, PI3K/AKT, NF‐kB), and activating the immune response. Bioactive compounds such as kaempferol, casticin, centaureidin, and chlorogenic acid derivatives contribute for showing selective activity against cancer cells while sparing normal cells. Some studies also highlight the toxic effect of *A. millefolium* L. on normal cells, although such evidence is limited. However, current research is largely limited to in vitro cell lines and small‐scale animal models. Further investigations are needed to explore in vivo tumor models, bioavailability, and comprehensive mechanistic effects in living organisms as well as contradictory findings in normal or non‐cancer cells to better assess its safety. The anticancer activity of *A. millefolium* L. is highlighted in Figure 5.

## 14 Reproductive Health

*Achillea millefolium* L. ethanolic extract was found to be effective in treating PCOS in a rat model. The 5 g/kg *A. millefolium* L. extract increased reproductive parameters, such as estrous cycles, ovarian morphology, and sex hormones, in a dose‐dependent manner. It also significantly reduced ovary and body weights (124.30 ± 3.23 mg and 240.33 ± 3.51 g) in PCOS rats compared to the control group (134.44 ± 5.72 mg and 295.66 ± 8.90 g). The number of cysts was increased in the PCOS group (9.0 ± 0.6), but 5 g/kg of *A. millefolium* L. reduced it (6.0 ± 0.7) (Hadijafari et al. 2021). Furthermore, a study was conducted to compare the effect of *A. millefolium* L. aqueous extract vaginal cream to the clotrimazole vaginal cream in women with vulvovaginal candidiasis. As a result, the 2% (w/w) extract cream showed a minimum inhibitory concentration of 37.5 mg/mL and improved symptoms in 53% of patients, while 1% clotrimazole was overall more effective (Zakeri et al. 2020).

Primary dysmenorrhea is characterized by menstrual pain without pelvic disease. It is caused by the excess production of prostaglandin, which leads to uterine hypercontraction, ischemia, and pain. It is a common gynecological issue among women of reproductive age that can impact daily activities, reduce quality of life, and cause psychological stress (Guimarães and Póvoa 2020). Herbal plants, drugs, and acupressure can reduce pain by decreasing prostaglandin levels, increasing beta‐endorphin levels, regulating nitric oxide, suppressing calcium channels, and improving uterine blood flow (Sharghi et al. 2019). According to a study, *A. millefolium* L. hydro‐alcoholic capsules (150 mg) significantly reduced pain scores (4.46 ± 1.53) in patients suffering from primary dysmenorrhea at the end of the first menstrual cycle, as compared to the M. chamomile group (5.29 ± 1.78). At the end of the second cycle, *A. millefolium* L. reduced pain levels (3.81 ± 1.06 vs. 4.33 ± 2.12), but the difference was not statistically significant (Radfar et al. 2019).

Currently, various modern, synthetic, and biotechnological drugs are being developed to expand treatment options. However, these advancements have also raised concerns about potential drug‐induced toxicities (Bai et al. 2025). Medicinal plants and their remedies play a protective role in treating chemical or drug‐induced organ toxicities, such as hepatotoxicity (Shakya 2020), renal toxicity (Rad et al. 2017), and reproductive toxicity (Ikuomola et al. 2024). Al‐Ezzy et al. (2019) determined the effect of high doses of methanolic extract (100 and 200 mg/kg) of *A. millefolium* L. on methotrexate‐induced sperm head abnormalities in male mice. As a result, 200 mg/kg extract reduced fertility and caused sperm abnormalities. A study was conducted to investigate the effect of an aqueous extract of *A. millefolium* L. on cyclophosphamide‐induced toxicity in rat testes. By results, 1.2 g/kg/day of extract elevated the body weight (194.83 ± 10.72 g), testes weight (1.69 ± 0.099 g), epididymides (1.00 ± 0.081 g), testosterone levels (above than 4 ng/mL), testicular antioxidant capacity (1.0 mmol·L^−1^/mg protein), relative organ weights, epididymal sperm count concentration and spermatogenic activities (Jalali et al. 2012). One other study found that 400 mg/kg of *A. millefolium* L. flower extract decreased body and epididymis weight (82/17 ± 7/96 g and 49/87 ± 2/22 (1000 × g)), sperm viability and motility (77/83% ± 3/11% and 74/83% ± 3/48% respectively), blood testosterone levels (445/5 ± 18/43 ng/dL), daily sperm production (DSP), epididymal sperm reserves (ESR) and overall percentage of fertility in male rats (Parandin and Ghorbani 2010). Dalsenter et al. (2004) investigated the effect of aqueous extract of *A. millefolium* L. leaf (0.3, 0.6, and 1.2 g/kg) on reproductive health in male rats for 90 days. It was reported that sperm number (255 ± 52.1 × 10^6^ in control and 251 ± 52.5 × 10^6^ in 1.2 g/kg of extract), body weight (367 ± 48.2 g in control and 376 ± 24.5 g in 1.2 g/kg of extract), and morphology didn't change significantly among groups. However, it was found that high doses of the extract increased the percentage of abnormal sperm (3.10% ± 0.41% at 1.2 g/kg of extract). In summary, *A. millefolium* L. may support reproductive health by improving ovarian function, reducing menstrual pain, regulating sex hormones, and protecting against drug‐induced reproductive toxicity. However, most of the evidence comes from animal studies or small‐scale human trials, with some studies also reporting negative effects at high doses such as reduced sperm quality and fertility. This highlights the need of more controlled clinical studies to establish safe dosing, long term efficacy and potential side effects in humans.

## 15 Antimicrobial and Antiparasitic Activity

As bacteria become increasingly resistant to antibiotics, it has become increasingly challenging to treat infectious diseases. However, in recent years, medicinal plants and their antimicrobial compounds have gained importance and are widely used because they have fatal effects on pathogenic bacteria (Eslammanesh et al. 2024).

A study in Bulgaria stated that *A. millefolium* L. essential oil (10 mg/mL) exhibited intermediate activity against *K. pneumoniae* (ATCC 13883) with an inhibition zone of 12 ± 1.41 mm, while showing weak activity against other microorganisms. No inhibition was detected against *A. niger* (ATCC 1015) and *A. flavus* (Nikolova et al. 2026). Similarly, Bilušić et al. (2025) reported that the aqueous herbal infusion of the whole plant of *A. millefolium* L. in Croatia did not exhibit any antibacterial activity against *E. coli* (ATCC 25922) and *S. aureus* (ATCC 25923), as no inhibition was observed at the tested concentrations (4–0.0078 mg/mL). The antibacterial and antifungal activities of *A. millefolium* L. essential oil from aerial parts were evaluated in Italy. The results showed that EO demonstrated the highest antibacterial activity against *Xanthomonas campestris* (8.9 ± 2.3 mm; 13.8 ± 1.9 mm; 19.3 ± 1.3 mm) and *Pseudomonas viridiflava* (4.3 ± 1.2 mm; 9.2 ± 1.3 mm; 17.7 ± 1.2 mm) at concentrations of 2500, 5000 and 10,000 ppm, respectively. As for antifungal activity, the highest inhibition was observed against *Penicillium italicum* (0.0 ± 0.0 mm; 20.9 ± 0.4 mm; 31.6 ± 0.8 mm) (Amato et al. 2025), Toplan et al. (2022) reported the antibacterial and anti‐candidal activities of different extracts (n‐hexane, chloroform and methanol), infusions and essential oils of *A. biebersteinii* and *A. millefolium* L. subsp. *millefolium* against various microorganisms in Turkey. Among all tested bacteria, the highest sensitivity was observed against *S. aureus* (ATCC 6538), with MIC values ranging from 0.125–1 μg/mL, whereas *Pseudomonas aeruginosa* (ATCC 27853) was highly resistant to both plant species, with MIC values exceeding 2 μg/mL in most tested forms. In terms of anti‐candidal activity, the strongest effect was observed in the n‐hexane and chloroform extracts of both plants, particularly against *C. utilis* (NRRL Y‐900) (MIC: 0.015–0.031 μg/mL), while infusions exhibited the weakest activity against *C. albicans* (ATCC 10231) and *C. tropicalis* (ATCC 750) with MIC values above 2 μg/mL.

More than 700 microbial species, including bacteria, fungi and others, are found in the human oral cavity. These microorganisms coexist through cooperative and competitive relationships; however, any disruption in oral conditions can disrupt this dynamic and lead to the development of infections and diseases (Du et al. 2022). A study investigated the antibacterial activity of aqueous, ethanolic, methanolic, acetone, and hydro‐ethanolic extracts of *A. millefolium* L. aerial parts on *Klebsiella*, *S. pyogenes*, and oral bacteria. The authors found that aqueous extracts at a concentration of 50 mg/mL had the highest antibacterial effect, with inhibition zones of 12 mm for *Klebsiella*, 12 mm for *S. pyogenes* , and 11 mm for oral bacteria. Minimum inhibitory concentration (MIC) and minimum bactericidal concentration (MBC) values were also found to be 50 mg/mL. Although the aqueous extract exhibited the strongest antibacterial effect compared to other extracts, the methanolic extract (also at 50 mg/mL) possessed antibacterial activity, albeit only against *S. pyogenes* (12 mm), with MIC and MBC values of the same magnitude (Fallah et al. 2020). Similarly, in another study, EO was extracted from the fresh leaves of *A. millefolium* L., yielding 0.4%. For its antimicrobial activity against four microorganisms (*C. albicans*, *S. epidermidis* , *E. coli* , *and K. pneumoniae*), EO was prepared as a 150 mg/mL solution and diluted serially. The results showed that EO had low antimicrobial activity, with MIC values above 1.5 mg/mL for all species. The results of the antimicrobial action tests showed a bacteriostatic profile (at 150 mg/mL for *S. epidermidis* and *E. coli* ), a bactericidal profile (at 150 mg/mL for *K. pneumoniae* ), and a fungistatic profile (≥ 37.5 mg/mL for *C. albicans*) (Daniel et al. 2020). These studies indicate the variation in the antimicrobial strength of *A. millefolium* L., likely due to differences in plant form used and their chemical composition. Fallah et al. (2020) evaluated different solvent extracts and stated that aqueous extract has stronger antimicrobial activity, while Daniel et al. (2020) investigated the essential oil, which exhibited mostly bacteriostatic or weak effects across the tested microorganisms.

Furthermore, according to a study conducted in Eastern Serbia, the essential oil of *A. millefolium* L. aerial parts exhibited strong antibacterial activity against the Gram‐positive bacteria *S. aureus* (ATCC25923) (MIC: 1.25 ± 0.10 μL/mL; MBC: 2.50 ± 0.17 μL/mL) while weaker activity was observed against the Gram‐negative bacteria *E. coli* (ATCC8739) (MIC: 20.00 ± 0.23 μL/mL; MBC: > 20 μL/mL). In contrast, *A. clypeolata* essential oil showed higher activity against *E. coli* (MIC: 10.00 ± 0.24 μL/mL; MBC: 20.00 ± 0.41 μL/mL) but comparatively lower activity against *S. aureus* (MIC: 2.50 ± 0.09 μL/mL; MBC: 5.00 ± 0.04 μL/mL) than *A. millefolium* L. (Aćimović et al. 2020). Antibacterial activity of *A. millefolium* L. ethanolic extract was determined against several strains of antibiotic‐resistant human pathogenic bacteria, including *K. pneumoniae* , *A. baumannii* , *E. coli* , and *P. aeruginosa* . The results showed that the extract inhibited the growth of *E. coli* and *A. baumannii* by 90% at a MIC value of 125 mg/mL. In comparison, it also reduced the growth of *K. pneumoniae* and *P. aeruginosa* by 90% at the MIC value of 250 mg/mL (Aliasghari et al. 2017). One study found that the inhibitory effect of the essential oil from the aerial parts of yarrow on various *Staphylococcus* species is dose‐dependent. For example, the inhibition zone diameter for *S. aureus* was shown to be 8.80 mm at a 10% concentration of *A. millefolium* L.; however, this inhibition increased to 10.14 mm at a 30% concentration. Similarly, the inhibition of *S. lentus* increased from 8.80 to 10.12 mm when the concentration of *A. millefolium* L. rose from 10% to 30% (Issabeagloo et al. 2012). Table 4 illustrates the antimicrobial activity of *A. millefolium* L. against various pathogens. It summarizes study locations, plant parts or form used, tested organisms, and reported MIC values or zone of inhibition.

**TABLE 4:** Antimicrobial activity of *A. millefolium* L.

Location	Part/Forms	Organism (s)	MIC/Zone of inhibition	References
Turkiye	Plant (methanol‐chloroform extract, later dissolved in methanol/50% aqueous methanol)	*E. coli* ATCC 25922, *B. subtilis* ATCC 11774, and *C. albicans*	Highest effect on *B. subtilis* (MIC: 0.162 mg/mL), moderate effect on *E. coli* (MIC: 3.125 mg/mL), no effect on *C. albicans* (MIC: 3.125 mg/mL)	(Baran et al. 2025)
Serbia	Flowering tops (herbs) (methanol and dichloromethane extract)	*L. monocytogenes, E. faecalis, S. aureus, S. aureus* MRSA, *S. flexneri, E. coli, S. enteritidis, A. baumannii, K. pneumoniae, P. mirabilis, P. aeruginosa, C. albicans*	Highest effect on *L. monocytogenes* (MIC: 1.25 mg/mL for both extracts) Good antifungal activity on *C. albicans* (MIC: 2.5 and 0.3125 mg/mL for methanol and dichloromethane extract, respectively)	(Cvetković et al. 2025)
Poland	Aerial Parts (hydro‐ethanolic extract)	*Candida albicans* ATCC10231, *Streptococcus agalactiae* PCM 2683, *Enterococcus faecalis* PCM 2784, *Proteus mirabilis* ATTC 29906, *Streptococcus mutans* ATTC 25175, *Staphylococcus epidermidis* ATTC 8853, *Streptococcus pyogenes* ATTC 19615, *Escherichia coli* UPEC PCM 176, *Enterococcus hirae* ATCC 10541, *Bacillus subtilis* PCM 486, *Staphylococcus aureus* 6538P, *Staphylococcus epidermidis* PCM 2118, *Escherichia coli* ATCC 8739, *Pseudomonas aeruginosa* PAO1, and *Ralstonia solanacearum* Z1	Highest activity shown against *Candida albicans, Pseuodomonas aeruginosa*, *Ralstonia solanacearum* and *Bacillus subtilis* (MIC: 2 mg/mL) Lowest activity for *Escherichia coli* UPEC and *Enterococcus faecalis* (MIC: 16 mg/mL)	(Michalak et al. 2025)
Turkey	EO	*Bacillus cereus NRRL B‐3711, Corynebacterium striatum ATCC BAA‐1293, Streptococcus sanguinis ATCC 10556, Staphylococcus aureus ATCC 700699*	Moderate to low activity shown *C. striatum* (MIC: 195 μg/mL) *B. cereus* (MIC:390 μg/mL), *S. sanguinis* (MIC: 780 μg/mL) and *S. aureus* (MIC: > 6250 μg/mL)	(Demirci et al. 2025)
Turkey	EO	*P. aeruginosa, S. typhimurium and E. coli*, *S. aureus, B. subtilis and E. faecalis*.	Shown highest zone of inhibition for *E. faecalis* (12–14 mm)	(Yildirim et al. 2023)
Spain	Inflorescences and upper dried leaves ethanolic extract YE, and its SAF fractions (YPF, YSF)	*H. pylori strains* (Hp48, Hp53, Hp59)	YSF showed highest effect (MIC:0.08 mg/mL) YPF (MIC: 0.08–0.14 mg/mL) YE (MIC: 0.14 mg/mL)	(Villalva et al. 2022)
Romania	Flowers hydro‐alcoholic extract	*Staphylococcus aureus, Streptococcus salivarius* and *Yersinia enterocolitica Candida albicans* and *Aspergillus niger*	Highest effect against *Staphylococcus aureus* (21 mm inhibition) Weaker effect on *Streptococcus salivarius* and *Yersinia enterocolitica* (10–11 mm inhibition) No antifungal activity shown	(Grigore et al. 2020)
Iran	EO	*S. aureus* and *P. aeruginosa*	MIC for both strains were 1 mg/mL	(Ghasemi et al. 2023)
France	Aerial parts EO	Bacteria: *B. cereus, St. aureus, B. subtilis, S. typhimurium, S. agona, St. epidermidis, S. enteritidis, E. coli* Fungi: *R. stolonifera, V. dahlia, C. gloeosporioids, B* *. cinerea* , *and A. niger*	Highest activity against *B. cereus* and *R. stolonifera* (MIC: 100 μg/mL and 1.6 mg/mL respectively)	(El‐Kalamouni et al. 2017)
Turkey	Aerial parts EO	Methicillin‐resistant *S. aureus* MRSA, *S. aureus* ATCC 6538, *B. cereus* CCM 99, *P. aeruginosa, E. coli* Q157:H7	Highest zone of inhibition against *S. aureus* MRSA (21 mm) While weakest against *P. aeruginosa* (10 mm).	(Sevindik et al. 2016)
Iraq (Mosul university)	Flower (ethanolic and aqueous extract)	*P. aeruginosa, S. enterica* (*Typhimurium*), *S. flexneri* , *M. luteus* , *S. aureus* and *E. faecalis* .	Ethanolic extract more effective; Maximum zone of inhibition showed for *P. aeruginosa* (30 mm)	(Hasson 2011)

Abbreviations: EO, essential oil; MIC, minimum inhibitory concentration; SAF, supercritical anti‐solvent fractionation; YE, yarrow extract; YPF, yarrow precipitated fraction; YSF, yarrow separator fraction.

Recent studies have also explored the effect of *A. millefolium* L. on various other microorganisms. For instance, a study was conducted in Poland to investigate the antibabesial activity of water (WE), ethanol (EE), and hexane/acetone (H/AE) extracts of *A. millefolium* L. aerial parts against *B. canis* . The results showed that all extracts at a concentration of 2 mg/mL exhibited inhibitory rates of 58.7% (±4.7%), 62.3% (±5.5%), and 49.3% (±5.1%) in an antibabesial assay (Guz et al. 2019). Similarly, the fumigant toxicity of *A. millefolium* L. and *P. ferulacea* against *S. granarius* and *S. oryzea* was determined in Turkey. The results revealed that *A. millefolium* L. caused a 98.85% mortality rate in *S. granarius* at 24 h and 100% mortality in *S. oryzae* (Şimşek et al. 2016).

Acaricidal activity of *A. millefolium* L. essential oil against two‐spotted spider mite ( *T. urticae* ) was demonstrated using leaf dipping and fumigation assays. The study concluded that *A. millefolium* L. essential oil was toxic to *T. urticae* , and a significant correlation was found between log concentration and mite mortality. According to the probit analysis, 24‐h‐LC50 values with 95% confidence limit of *A. millefolium* L. for leaf dripping bioassay were 1.208% v/v (0.957%–1.590% v/v), and as for fumigation bioassay, they ranged from 1.801 (1.524–2.213) to 3.586 (3.101–4.367) μl/l air respectively (Ebadollahi et al. 2016). In Iran, a study was performed to investigate the anti‐leech activity of *A. millefolium* L. ethanolic extract against *L. nilotica* . The study found that at a dose of 600 mg, the ethanol extract exhibited a 3+ anti‐leech effect (killing each leech within 61–120 min), whereas the standard drugs such as Levamisole and Niclosamide showed a stronger 4+ anti‐leech activity (killing leeches in 1–60 min) (Bahmani, Abdi, et al. 2014; Bahmani, Zargaran, and Rafieian‐Kopaei 2014).

The antiviral activity of *A. millefolium* L. and *T. vulgaris* extracts was evaluated against Newcastle Disease Virus (NDV) in embryonated eggs. The study demonstrated that both extracts significantly reduced viral effects by 56‐fold or more, with the maximum non‐toxic concentrations for *A. millefolium* L. and *T. vulgaris* being 10 μg/mL and 50 μg/mL, respectively (Rezatofighi et al. 2014). Similarly, the effectiveness of *A. millefolium* L., *T. vulgaris*, and propolis hydro‐alcoholic extracts was also assessed for the treatment of cutaneous leishmaniasis in BALB/c mice. The study demonstrated that these extracts led to a reduction in the mean ulcer size in the treated groups (43.29%, 36.09%, and 43.77%, respectively), showing their potential in treating this condition (Nilforoushzadeh et al. 2008). According to these studies, it can be concluded that *A. millefolium* L. displays a broad spectrum of antimicrobial and antiparasitic activities, showing inhibitory effects against various bacteria, fungi, viruses, and parasites. However, the reported efficacy is highly inconsistent, varying with extract type, concentration, and the organism tested, with some pathogens showing minimal or no response. In many cases, activity is only observed at higher doses, along with lower efficacy compared to standard drugs. Most findings come from in vitro assays or experimental models, highlighting the need for clinical validation, dose optimization, and better identification of bioactive components responsible for this effect.

## 16 Miscellaneous

*Achillea millefolium* L. also has other beneficial effects that are evident through scientific studies. The wound‐healing properties of *A. millefolium* L. ointments (hydro‐alcoholic extract) have been reported in primiparous women, and it is found that *A. millefolium* L. ointment (5% weight ratio in a 30 g tube) alleviated the symptoms such as redness, edema, ecchymosis, pain (*p* < 0.001), and helped in episiotomy wound healing and recovery. However, no significant difference was found for wound dehiscence and discharge between groups (Hajhashemi et al. 2018). Mahmoudi et al. (2023) reported that an ointment containing 5% of hydro‐alcoholic extract of *A. millefolium* L. was effective for treating grade 1 and 2 hemorrhoids. This study found that the ointment alleviated symptoms such as pain (1.11 ± 1.83), discomfort after defecation (1.79 ± 1.95), and the intensity and frequency of bleeding in patients by 86%, and reduced itching by 93%. Another study by Hashemian et al. (2021) also demonstrated that a 1% *A. millefolium* L. ointment was effective against recurrent idiopathic epistaxis. This ointment was administered to the case group, and after 3 months of treatment, the severity score for epistaxis (0.20 ± 0.43), bleeding frequency (0.28 ± 0.46 days per week), and duration of bleeding (0.04 ± 0.20 days) were reduced in patients, with no adverse side effects.

Another study investigated the effect of *A. millefolium* L. hydro‐alcoholic extract on cisplatin‐induced ocular toxicity and reported modulation of inflammatory markers. A 400 mg/kg dose increased IL‐10 levels (greater than 150 pg/mL) while decreasing IL‐1β levels (< 80 pg/mL). Additionally, expression levels of NF‐κB, Caspase‐3 mRNA, and TNF‐α mRNA were also reduced to 1.5‐ and 1.2‐fold changes (Okkay et al. 2021). Ethanolic extract derived from *A. millefolium* L. flowers considerably reduced vascular injuries in heart (edema: 1.21 ± 0.31, congestion: 1.25 ± 0.50), liver (Dilatation of sinusoid: 0.50 ± 0.58, inflammatory cells infiltrations: 0.00 ± 0.00), and kidney (glomeruli diameter: 124.15 ± 6.72, bowman's space: 20.69 ± 2.25) induced by cisplatin, thus showing their potential as protective agents against chemotherapy induced organ damage (Eslamifar and Sabbagh 2020).

Chemotherapy is a common cancer treatment that targets cancer cells but can also damage normal cells, leading to side effects that range from nausea and hair loss to serious infections, organ damage, and severe oral mucositis (Juthani et al. 2024). Oral mucositis (OM) is a common adverse effect caused by chemotherapy or head and neck radiotherapy. This condition results in painful, erosive, or ulcerative lesions in the mouth, causing mild to severe discomfort. These mucosal injuries can reduce the quality of life by affecting nutrition, increasing the risk of infections, and prolonging hospital stay (Curra et al. 2018). According to a study, *A. millefolium* L. mouthwash was found to be effective against oral mucositis caused by chemotherapy in patients with acute myeloid leukemia. The effect was particularly shown on the 10th and 20th days of treatment. The case group showed an improvement in oral mucositis grade (0.50 ± 0.52), experienced mild mucositis, decreased pain, and lower consumption of painkillers at the 20th day of treatment (Hajisalem et al. 2019). Miranzadeh et al. (2015) demonstrated that a mixture of mouthwash and *A. millefolium* L. distillate (in a 50:50 ratio) remarkably reduced oral mucositis caused by chemotherapy. In the experimental group, mean severity scores significantly reduced from 1.07 ± 0.85 after 7 days to 0.32 ± 0.54 after 14 days, as compared to the control group. Both studies indicate that *A. millefolium* L. has beneficial effects against chemotherapy‐induced oral mucositis. However, differences in outcomes have been observed, which may be due to variations in formulation methods, concentrations, and treatment duration. One study highlighted the role of *A. millefolium* L. mouthwash alone in gradual improvement over a longer period, whereas the other study reported a faster and pronounced reduction when mouthwash was used in combination with distillate.

*Achillea millefolium* L. was also found to be neuroprotective as its aqueous extract at a dose of 7 mg/kg of extract restored memory function and also reduced anxiety behaviors in ovariectomized female Wistar rats with cerebral ischemia (Jahromi et al. 2019). A study evaluated the use of *A. millefolium* L. extract in olive and sunflower oil to treat skin irritation. The skin irritation was induced using the sodium lauryl sulfate test, and then the extract was applied for 7 days. According to the results, extract‐based oils reduced skin irritability, improved skin hydration, and showed moisturizing and anti‐inflammatory effects. However, there was no significant difference in the activity of the oils (Tadić et al. 2017). *A. millefolium* L. efficacy against radiation‐induced dermatitis in breast cancer patients was also investigated. The *A. millefolium* L. cream reduced complications in the skin compared to the placebo, but the difference was not statistically significant, and both the case and control groups had the same levels of grade three toxicities (7.7% and 7.1%, respectively) (Malekzadeh et al. 2016).

A study was conducted to assess the potential of *A. millefolium* L. (50, 250, 500, and 1000 mg/kg) on improving cognitive abilities and memory recognition in mice using the novel object recognition test. As a result, it was found that there was no statistically significant difference in object exploration and activity levels between the extract‐treated and control groups (*p* > 0.05). It was also found that the extract played no particular role in impairing memory function (Ayoobi et al. 2013). Moreover, *A. millefolium* L., along with other herbs (*Eleutherococcus*, *A. millefolium* L., *and L* *. album* ), was also evaluated for its effects on atopic dermatitis. The groups treated with this tri‐herb combination and the control group both showed decreased SCORAD grades (31.8 ± 2.8 after the 2nd week), and affected body surface area (23.6 ± 4.4 at 8 weeks) (Shapira et al. 2005). According to these studies, *A. millefolium* L. possesses diverse beneficial effects, including hepatoprotective, wound healing, neuroprotective, antioxidant and anti‐inflammatory effects. However, the findings are inconsistent, as some studies report improvements while others show limited or no effects. Most studies have small sample sizes, short durations or use pre‐clinical models, so results are unclear. Therefore, well‐designed and large‐scale clinical trials are needed to validate its safety and efficacy.

## 17 Synergism and Drug Interactions

Different medicinal plants can act synergistically to provide better health benefits. As a result, the effect of their combination is greater and more effective than the individual activity of each plant. One study determined and compared the synergistic and antagonistic effects of the antimicrobial activity of methanolic and diethyl ether extracts of *A. millefolium* L., *A. cretica*, *C. intybus* , *E. seguieriana* , *and H. perforatum* against different Gram‐positive and Gram‐negative bacteria. The results showed that the methanol extract mixture of *A. millefolium* L. with *C. intybus* showed strong synergism against Gram‐positive bacteria (FIC: 0.49). *In contrast*, only partial synergism was observed for Gram‐negative bacteria (FIC: 0.64–0.67). Similarly, the methanol extract mixture of *A. millefolium* L. with *H. perforatum* (FIC: 0.48–0.49) and the diethyl ether extract mixture of *A. millefolium* L. and *H. perforatum* for Gram‐positive (FIC: 0.24–0.25) showed synergistic effects and exhibited notable antimicrobial activity. *A. millefolium* L. also exhibited a moderate synergistic effect with *A. cretica* in methanol extract (FIC: 0.57–0.75) (Doğan and Darcan 2022). *A. millefolium* L. has also shown pain‐relieving effects when combined with other plants. According to one study, an extract mixture of *A. millefolium* L. and *O. vulgare* , encapsulated in liposomes, was administered to the rats to investigate their anti‐nociceptive synergistic effects. In the results, it was found that this mixture had produced strong pain‐relieving effects (66%) (Hassanzadeh‐Kiabi and Negahdari 2018).

*Achillea millefolium* L. has also been used in combination with *G. glabra and M. chamomilla* to improve gut health in patients suffering from irritable bowel syndrome. After 4 weeks of intervention, the authors found reduced abdominal pain in 70.6%, incomplete excretion in 64.7%, mucus excretion in 92.2%, and bloating in 68.6% patients as compared to the control group (19.6%, 35.0%, 66.7% and 27.5% respectively) (Rahimi et al. 2023). Kazemian et al. (2017) reported that a 3‐month intervention with a herbal mixture of *A. millefolium* L., *B. carterii*, *and Z. officinale* improved bloating severity in patients (24.25 ± 19.28 in the case group compared to 54.31 ± 17.68 in the control group), and also led to satisfactory bowel habits (64.5 ± 19.80; 49.31 ± 15.83). Similarly, there was an improvement in quality of life (QOL) in both groups (54.0 ± 27.93; 58.18 ± 18.16). The QOL improved more in men from both case and control groups, while no significant changes were observed in women. According to a study on antidiabetic activity by Mir et al. (2018), it was shown that a mixture of *A. millefolium* L. and *T. polium* significantly improved HDL levels (up to 35 mg/dL) and body weight (193 ± 15 g as compared to 162 ± 18 g in diabetic control) while it also significantly reduced serum ALP levels (≈600 U/L as compared to ≈1200 U/L in diabetic control) in STZ‐induced rats. Shapira et al. (2005) conducted a study to determine the effect of tri‐herb combination on atopic dermatitis. The treatment group received a mixture of *Eleutherococcus*, *A. millefolium* L., and *L. album* , while the control group was given a placebo. As a result, both groups showed a reduction in basal scores (6.0 ± 1.0 after 2 weeks), relapse rates (7.7 ± 1.0 at the 4th week), and mean intensity scores (5.8 ± 0.5 at 8 weeks). But there was no significant difference between these groups.

*Achillea millefolium* L. has also been compared with other plants of the same and different species to investigate its antioxidant and antimicrobial potential. A study was conducted by Stanojević et al. (2022) to determine and compare the antioxidant potential of *A. millefolium* L. and *H. arenarium*. As a result, the authors found that *A. millefolium* L. demonstrated better antioxidant activity than *H. arenarium* in the ABTS assay (26.03 mg/cm^3^ vs. 88.52 mg/cm^3^ ABTS radical). However, in the Ferric Reducing Antioxidant Power (FRAP) assay, *H. arenarium* showed higher reducing power (7.16 mg Fe^2+^/g vs. 5.72 mg Fe^2+^/g). The antimicrobial activity of *A. millefolium* L. is also compared with that of other plants. In one study, the antimicrobial activity of hexane, ether, and methanol extracts of the aerial parts of *A. clavennae* , *A. holosericea* , *A. lingulata* , *and A. millefolium* L. was evaluated against different bacterial strains. As a result, it was found that *A. clavennae* performed the highest antimicrobial activity (27 mm inhibition zone for *S. aureus* , and 22 mm for *P. aeruginosa* ) compared to the others, while *A. millefolium* L. also exhibited significant activity (16 mm for *S. aureus* , and 17 mm for *P. aeruginosa* ) (Stojanović et al. 2005).

*Achillea millefolium* L. also interacts with other drugs, either by enhancing their effects or by slowing down their performance. *A. millefolium* L. can help in better blood circulation and lower blood pressure, and can make these blood pressure medications work better. The blood pressure‐regulating effect of *A. millefolium* L. may be attributed to the presence of chemical constituents such as achillin and leucodin, which aid in the relaxation of blood vessels. These chemicals are responsible for releasing nitric oxide and increasing the production of cGMP, a molecule that helps in the relaxation of smooth muscles in blood vessels. This results in the regulation of blood pressure to normal levels. Although *A. millefolium* L. can be beneficial, it should not be taken with blood pressure medication, certain antiplatelet drugs, or sedatives, as it can cause nausea, dizziness, and vomiting (Csupor et al. 2019). *A. millefolium* L. has anticoagulant or blood thinning activity but it can increase the risk of bleeding when taken together with anticoagulant drugs like aspirin, ibuprofen, and warfarin etc. *A. millefolium* L. also interferes with the breakdown of drugs or slows down the action of drugs like diazepam, paracetamol etc. as it contains chamazulene which suppresses the actions of CYP1A2 and CYP3A4, the main enzymes present in the liver. As a result, drug levels increase in the body, and there is a greater chance of their side effects and toxicity. *A. millefolium* L. also interferes with photo‐dermatitis drugs and can cause skin irritability, skin sensitivity, and skin burn. A recently identified compound, α‐peroxyachifolid, may be responsible for these conditions (Farasati Far et al. 2023). Although, studies on drug interaction of *A. milefolium* L. are limited, several of its bioactive compounds present have been reported to interact with drugs. Luteolin, in combination with gefitinib has shown anticancer activity against PC‐3 prostate cancer cells via inhibition of EGFR and GAK kinase activity, resulting in reduced cell viability. Additionally, it induces miR‐630 expression, which promotes cell cycle arrest and inhibits cancer progression (Çetinkaya and Baran 2023) Apigenin exhibits positive synergistic interaction with aspirin by enhancing anticancer activity in colon cancer cells through suppression of NF‐κB and COX‐2 expression, leading to reduced cell proliferation. In contrast, it shows a negative interaction with venlafaxine by inhibiting liver microsomal CYP450 isoenzymes, resulting in reduced drug metabolism, increased plasma concentration and systemic exposure (Li et al. 2023). Kaempferol inhibits P‐glycoprotein (P‐gp) activity, which isresponsible for limiting drug absorption and promoting drug elimination, thereby increasing the bioavailability of its substrate drugs such as tacrolimus and paclitaxel, which may elevate the risk of harmful effects and toxicity (Niziński et al. 2025). Quercetin inhibits CYP2C9 enzyme, which normally converts diclofenac into its more active metabolite form, 4′‐hydroxy‐diclofenac. This results in reduced bioavailability of this active metabolite, making the combination of quercetin and diclofenac unsuitable for pain management (Rivas García et al. 2024). Taken together, *A. millefolium* L. exhibits synergism with several medicinal plants. However, results are inconsistent across studies, as outcomes vary depending on plant combinations, doses, and extract types. Drug interactions such as blood thinning effects, interference with liver enzymes and drug metabolism are reported but remain underexplored with limited scientific evidence. Therefore, well‐designed clinical studies are needed to better understand these synergistic effects, assess safety and toxicity and clarify underlying mechanisms. Additionally, more evidence‐based data are required to evaluate its herb‐drug interaction, its effects on drugs metabolism, efficacy and overall impact on the body.

## 18 Biomedical Applications of *A. millefolium* L.‐Derived Nanoparticles

Herbal medicines offer health and cosmetic benefits with fewer side effects, as their active ingredients work synergistically. However, herbal drugs also face some challenges, such as poor stability, low absorption, and low bioavailability, which reduce their effectiveness. These limitations have led to the development of novel drug delivery systems (NDDS), which employ techniques such as phytosomes, liposomes, emulsions, and nanoparticle formulations to enhance the stability, absorption, and therapeutic efficacy of these drugs (Pagar and Khandbahale 2019). Nanotechnology is involved in the development of small structures, with sizes ranging from 1 to 100 nm. Physical, chemical, and biological methods can be used to synthesize nanoparticles. Biological methods utilize medicinal plants and microorganisms to produce nanoparticles. Nanoparticles made from medicinal plants and their extracts are already being used for various beneficial purposes (Alharbi et al. 2022).

The nanoparticles made from *A. millefolium* L. are known to have various health applications. According to one study, zinc nanoparticles (Zn NPs) derived from *A. millefolium* L. exhibit cardioprotective activities. These nanoparticles inhibited *NF‐κB* signaling and increased PPAR‐γ expression. In the study, these particles also reduced inflammatory markers, such as IL‐6, IL‐1β, and TNF‐α, in the mice hearts. In animals administered Zn NPs, ST‐segment depression was also effectively prevented. The regulation resulted in reduced mortality in rats and improved cardiac condition (Li et al. 2024). Another study also investigated the in vitro cytotoxic effects of zinc oxide (ZnO) nanoparticles synthesized from the aqueous extract of *A. millefolium* L. Human cancer cell lines A549 (lung) and HT29 (colon) were used in this study. The nanoparticles were tested at concentrations ranging from 0 to 400 μg/mL. The nanoparticles exhibited IC50 values of 46.47 μg/mL for A549 cells and 42.82 μg/mL for HT29 cells, respectively. According to this study, the particles had strong and dose‐dependent cytotoxic activity (Acar 2021).

Chitosan *A. millefolium* L. nanoparticles (AMCSNPs) have demonstrated notable antibacterial activity. In one study, AMCSNPs exhibited antibacterial activity against both Gram‐positive *B. subtilis* and Gram‐negative *P. aeruginosa* and showed a maximum zone of inhibition against them (30 ± 0.5 mm). These nanoparticles exhibited three times the activity of *A. millefolium* L. alone. Moreover, these particles were also responsible for anti‐urolithiatic activity, showing 68% inhibition in the aggregation assay and 51.26% inhibition in the nucleation assay (Kain and Kumar 2020). In another study, *A. millefolium* L. essential oil and its chitosan‐based nanoparticles (prepared at different pH levels: 3.5, 4.5, and 5.5) were investigated to determine their effects against a common pest, *T. urticae*. According to 24‐h fumigation LC50 values, free EO had high toxicity, while the toxicity of nanoparticles decreased with increasing pH (5.05 μL/L air (EO), 18.90 μL/L (pH 3.5), 60.28 μL/L (pH 4.5), and 127.4 μL/L (pH 5.5) respectively). As for 48‐h contact toxicity, encapsulated particles were more effective and toxic (LC50: 5.51 μL/cm^2^ (EO), 4.23 μL/cm^2^ (pH 3.5), 3.30 μL/cm^2^ (pH 4.5), and 1.56 μL/cm^2^ (pH 5.5)). Although particles prepared at a pH of 5.5 had the lowest fumigant activity, they showed the highest contact toxicity. Additionally, these nanoparticles demonstrated acaricidal activity for up to 24 days, compared to the free EO, which showed this activity only for up to 9 days (Ahmadi et al. 2018). Both studies show varied biological effects of *A. millefolium* L. chitosan nanoparticles. One study reported their pronounced antimicrobial and anti‐urolithiatic effects, while the other demonstrated variable acaricidal toxicity, depending on pH, exposure time. These differences may be due to variation in formulation conditions, pH dependency or the type of organisms tested.

In one study, an aqueous extract of *A. millefolium* L. was used for the green synthesis of silver nanoparticles (Ag NPs) to investigate its antimicrobial activity against *E. coli* , *Fusarium*, and *A. niger*, as well as its cytotoxic activity against MOLT‐4 acute lymphoblastic leukemia cell line. By results, the particles showed strong antimicrobial activity against the tested *E. coli* in agar well diffusion and disc diffusion methods (15 ± 0.2 and 13 ± 0.1 mm), *Fusarium* (11 ± 0.1 and 10 ± 1.1 mm), and *A. niger* (9 ± 0.2 mm, disk diffusion only). The Ag NPs were also highly cytotoxic against the cancer cell lines, even more effective than cisplatin (IC50: 0.011 μm vs. 1.7–1.8 μm) (Karimi and Mahdavi Shahri 2021). In another study, aqueous, ethanol, and methanol extracts of *A. millefolium* L. were used to synthesize silver nanoparticles, and their antimicrobial activity was investigated. Both nanoparticles and extracts exhibited antimicrobial activity against both Gram‐positive ( *S. aureus* , *B. subtilis* ) and Gram‐negative ( *S. enterica* , *E. coli* , *P. aeruginosa* ) bacteria. However, nanoparticles made from the methanol extract showed the highest activity compared to the others (*S. aureus*: 14.33 ± 0.33 mm and *P. aeruginosa*: 13.67 ± 0.33 mm). Similarly, these methanol‐based particles also exhibited notable antioxidant activity in DPPH radical scavenging assays (IC50: 7.03 ± 0.31 μg/mL) (Yousaf et al. 2020). Overall, nanoparticles made from *A. millefolium* L. show promising therapeutic potential, including heart protection, anticancer and antimicrobial activity etc. However, current research is mostly limited to laboratory, in vitro or animal model settings. Variability in nanoparticle type, synthesis method, and doses can lead to inconsistent findings. Therefore, detailed investigations on safety, efficacy, in vivo effects, and underlying mechanisms of these nanoparticles are needed.

## 19 Commercial Applications

*Achillea millefolium* L. is increasingly gaining recognition in industrial and commercial sectors. The products derived from *A. millefolium* L. are particularly employed in tea mixtures and phytopharmaceuticals. This extensive utilization is attributed to bioactive components, which offer remarkable pharmacological properties. Due to its phytochemical constituents, this plant holds significant importance in various industries (Vladić et al. 2020).

Scientific data indicate that *A. millefolium* L., alongside other Achillea species, is commonly used in the food industry to improve the quality of food and ensure the safety of food products (El‐Kalamouni et al. 2017). These species are effective in preserving food due to their antioxidant, antimicrobial (Benali et al. 2020), and advanced glycation end products (AGE) inhibitory effects (Afshari et al. 2018). Moreover, *A. millefolium* L. has also been incorporated in the production of functional food, especially kombucha beverages. These *A. millefolium* L.‐enriched kombucha drinks have shown antifungal and antibacterial advantages against *E. coli* , *S. aureus* , *B. subtilis* , *K. pneumoniae* , *P. vulgaris* , *P. mirabilis* , *A. niger* , *and C. albicans*. These beverages also exhibit antitumoral and antioxidant activities, highlighting their potential as beneficial functional foods (Vitas et al. 2018; Salehi et al. 2020). In the European Union, aerial parts of *A. millefolium* L. are valued for their use as food supplements (Mannila et al. 2023). In addition, herbal teas and infusions prepared from *A. millefolium* L. aerial parts are used for calming, digestive and detoxification purposes. It is also incorporated in functional beverages, combined with other herbs for its gut health support and anti‐inflammatory effects. In Europe, its extracts are added in fortified water, highlighting its antioxidant value (Prisa and Jamal 2025). *A. millefolium* L. essential oils are incorporated into tinctures and liqueurs, its flowers are used for making tea, while its bitter and spicy young leaves can be used as flavoring agent in sauces, fish or soups (Konarska et al. 2023).

The idea of functional and nutraceutical foods has expanded new directions in nutritional research. Now‐a‐days, food is not only just considered as a source of nutrition for consumers but also a way to improve health (Butt and Sultan 2010). Nutraceuticals are food‐based products that provide both nutrition and therapeutic benefits, including dietary supplements, functional foods, vitamins, and herbal products. They contain bioactive components that support physiological functions and may help prevent and manage diseases. Herbal nutraceuticals are derived from plant parts such as flowers, leaves, roots, seeds and are widely used to improve health and reduce diet‐related diseases. *A. millefolium* L. is a medicinal herb containing compounds like azulene, caryophyllene, borneol, thujone and is traditionally used for treating cold, fever, loss of appetite, digestive disorders and menstrual disorders (Bommakanti et al. 2023). In a clinical trial, 500 mg/day *A. millefolium* L. capsules improved metabolic health in type 2 diabetic patients by reducing liver enzymes like ALT (24.41 ± 6.84 IU/L) and AST (18.76 ± 6.77 IU/L) while also improving lipid profile parameters such as LDL (80.86 ± 32.10 mg/dL), TGL (147.3 ± 66.79 mg/dL), and TC (150.1 ± 38.77 mg/dL) (Daneshvar‐Ghahfarokhi et al. 2025). In a recent in vivo study, supplementation with non‐encapsulated phenolic‐rich ethyl acetate fraction (PRF) and its nano‐encapsulated form (PRF‐NLs) (10 mg/TPC/kg/BW/day) derived from *A. millefolium* L. resulted in improved body weight gain (0.15 and 0.17 g), feed intake (3.1 and 3.3 g), and decreased liver enzymes such as ALP (243.0 and 228.7 IU/L), SGOT (143.1 and 135.6 IU/L) and SGPT (139.4 and 126.5 IU/L) (Nateghi et al. 2022). In another study, hydro‐alcoholic extract of *A. millefolium* L. exhibited appetite‐stimulating effects in Wistar rats. It was revealed that 50 and 100 mg/kg doses significantly elevated energy intake (58.5 ± 0.12 and 61.8 ± 0.23 kcal), but ghrelin levels were reduced after 1 h of a 100 mg/kg dose (*p* < 0.05) (Nematy et al. 2017). Furthermore, *A. millefolium* L. powder tea (4 g) has reduced average pain scores in primary dysmenorrhea patients after 1 and 2 months of treatment (1.02 ± 0.84 and 1.59 ± 1.81, respectively) (Jenabi and Fereidoony 2015). These findings across different formulations (capsules, extracts, and herbal tea) highlight *A. millefolium* L. potential as nutraceutical agent however more clinical‐based studies are needed to validate its safety and efficacy.

**FIGURE 6:**
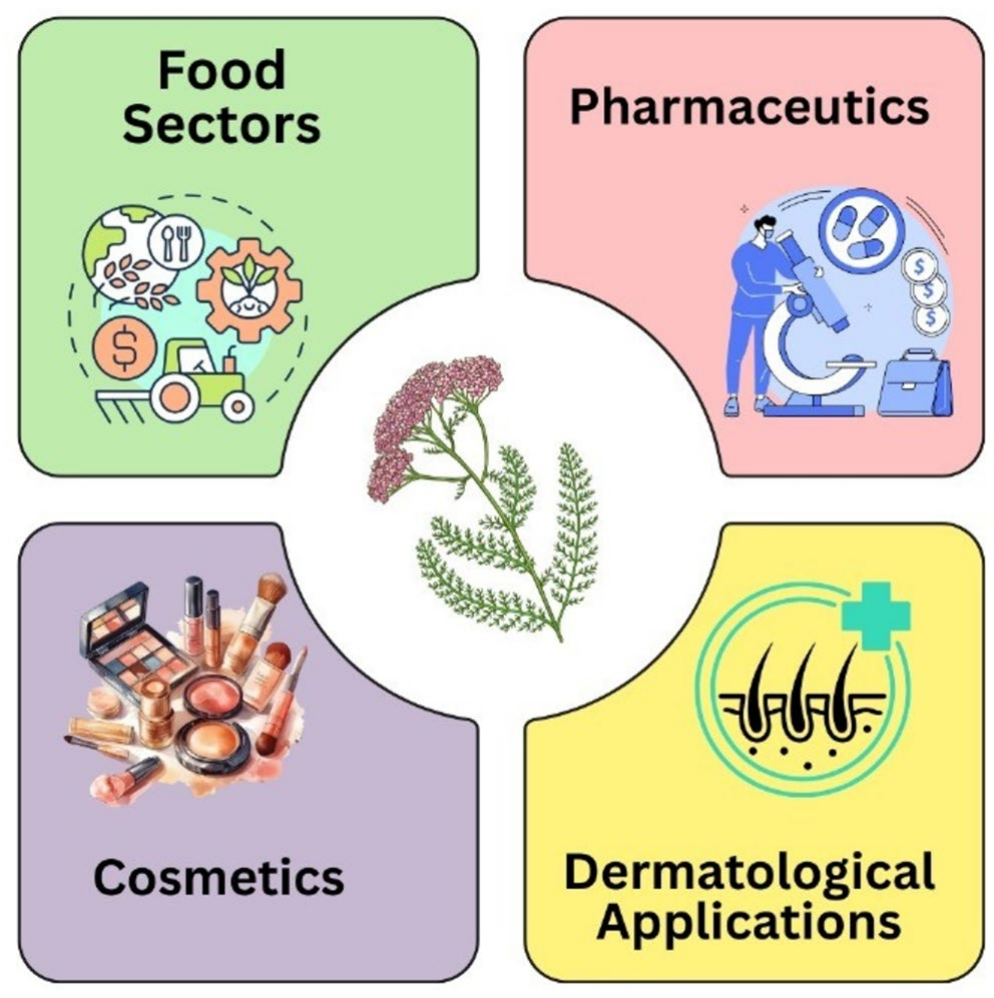
Commercial Applications of *Achillea millefolium* L.

*Achillea millefolium* L. derived materials are extensively used in cosmetics, industry, where extracts and oils are incorporated in products such as lotions, masks, and creams. These components play various roles including moisturizing, conditioning, and soothing and are also used in the management of skin burn and inflammation (Konarska et al. 2023). Distillates, juices, and essential oils made from *A. millefolium* L. are futher utilized due to their skin‐nourishing, wound‐healing, antioxidant, and cooling properties. The alcoholic and hydro‐alcoholic extracts have significant dermatological applications due to their anti‐inflammatory, skin‐lightening, and other activities (Gaweł‐Bęben et al. 2020). Furthermore, other extracts, such as those made with hexane, ethanol, and ethyl acetate, have photoprotective properties, meaning that high concentrations of bioactive components can be extracted from these solvents, which have protective effects. *A. millefolium* L. ethanol and ethyl acetate extracts have high SPF values, UVA, and UVB absorption that mark their potential as protective ingredients in sunscreen formulations. Thus, cosmetics containing *A. millefolium* L. extracts can be considered safe (Berbatovci‐Ukimeraj et al. 2024). All in all, commercial applications of *A. millefolium* L. are expanding due to its pharmacological and functional properties. Its use in functional food, beverages, and skin care formulation highlights its versatility and consumer acceptance. However, most evidence comes from in vitro studies and small‐scale product development and limited standardization. Further research is needed to critically evaluate product composition; its stability, safety, and efficacy in clinical settings, with long term effects and possible side effects. The applications of *A. millefolium* L. are mentioned in Figure 6.

## 20 Conclusion and Future Perspectives

*Achillea millefolium* Linn., commonly known as yarrow, is an extraordinary medicinal plant found worldwide and known by various local names. It contains compounds like flavonoids, phenols, alkaloids, and essential oils. The therapeutic potential of its leaves, flowers, and aerial parts is evident through scientific data. Traditionally, it has been used extensively due to its anti‐inflammatory, liver‐protective, and other beneficial properties. In addition, it possesses pharmacological properties, including antidiabetic, antioxidant, antimicrobial, and gastroprotective effects, as well as wound‐healing properties. *A. millefolium* L. works synergistically with other plants and interacts with other drugs or medications, either enhancing or inhibiting their action. *A. millefolium* L. has also been utilized in the synthesis of nanoparticles and is incorporated in the food and cosmetic industries. However, some limitations exist, as studies on its non‐volatile constituents and evidence on its plant–drug interactions remain insufficient. In addition, contradictory findings across different activities in current literature are relatively few. This highlights the need for systematic and evidence‐based studies to characterize its non‐volatile compounds, evaluate possible adverse or toxic effects and understand its correlation with modern drugs. In the future, research on *A. millefolium* L. should also focus more on identifying and isolating its primary bioactive components for further use in pharmaceuticals. Clinical trials are necessary to support its traditional uses and to explore its potential in the medical field. Furthermore, its potential to improve the effectiveness of drugs and alleviate side effects through herb‐drug interaction must be investigated thoroughly. As *A. millefolium* L. is used in the development of nanoparticles, the application of these formulations for various chronic diseases should be studied. Its use in the food and cosmetic fields should be expanded by conducting more safety and efficacy trials, as well as regular testing of new products.

## Author Contributions

**Sabrin R. M. Ibrahim:** supervision, writing – review and editing. **Tooba Majeed:** conceptualization, writing – original draft. **Muhammad Tauseef Sultan:** writing – original draft, conceptualization. **Ahmad Mujtaba Noman:** writing – review and editing, methodology, investigation. **Hagar M. Mohamed:** data curation, investigation, resources. **Mohamed A. Abdelgawad:** writing – review and editing. **Entessar Al Jbawi:** supervision, investigation, data curation. **Hassan Raza:** methodology, software, data curation. **Gamal A. Mohamed:** investigation, data curation. **Nimra Anees:** writing – review and editing. **Muzzamal Hussain:** supervision, investigation, writing – review and editing. **Samy Selim:** investigation, methodology. **Ehab M. Mostafa:** writing – review and editing. **Ayman Salama:** resources, validation, visualization. **Muhammad Imran:** validation, visualization, resources.

## Funding

The authors have nothing to report.

## Ethics Statement

This research is not submitted elsewhere and is not under consideration in any journal. The study does not involve any human or animal testing.

## Consent

All authors are willing to publish this manuscript. All the co‐authors are willing to participate in this manuscript.

## Conflicts of Interest

The authors declare no conflicts of interest.

## Data Availability

The data that support the findings of this study are available from the corresponding author upon reasonable request.
